# Unraveling Antimicrobial Resistance Genes and Phenotype Patterns among *Enterococcus faecalis* Isolated from Retail Chicken Products in Japan

**DOI:** 10.1371/journal.pone.0121189

**Published:** 2015-03-17

**Authors:** Arata Hidano, Takehisa Yamamoto, Yoko Hayama, Norihiko Muroga, Sota Kobayashi, Takeshi Nishida, Toshiyuki Tsutsui

**Affiliations:** 1 Viral Disease and Epidemiology Research Division, National Institute of Animal Health, National Agriculture, and Food Research Organization, Tsukuba, Ibaraki, Japan; 2 EpiCentre, Institute of Veterinary, Animal and Biomedical Sciences, Massey University, Palmerston North, New Zealand; 3 Animal Products Safety Division, Food Safety and Consumer Affairs Bureau, Ministry of Agriculture, Forestry and Fisheries, Chiyoda-ku, Tokyo, Japan; U. S. Salinity Lab, UNITED STATES

## Abstract

Multidrug-resistant enterococci are considered crucial drivers for the dissemination of antimicrobial resistance determinants within and beyond a genus. These organisms may pass numerous resistance determinants to other harmful pathogens, whose multiple resistances would cause adverse consequences. Therefore, an understanding of the coexistence epidemiology of resistance genes is critical, but such information remains limited. In this study, our first objective was to determine the prevalence of principal resistance phenotypes and genes among *Enterococcus faecalis* isolated from retail chicken domestic products collected throughout Japan. Subsequent analysis of these data by using an additive Bayesian network (ABN) model revealed the co-appearance patterns of resistance genes and identified the associations between resistance genes and phenotypes. The common phenotypes observed among *E*. *faecalis* isolated from the domestic products were the resistances to oxytetracycline (58.4%), dihydrostreptomycin (50.4%), and erythromycin (37.2%), and the gene *tet*(L) was detected in 46.0% of the isolates. The ABN model identified statistically significant associations between *tet*(L) and *erm*(B), *tet*(L) and *ant*(6)-Ia, *ant*(6)-Ia and *aph*(3’)-IIIa, and *aph*(3’)-IIIa and *erm*(B), which indicated that a multiple-resistance profile of tetracycline, erythromycin, streptomycin, and kanamycin is systematic rather than random. Conversely, the presence of *tet*(O) was only negatively associated with that of *erm*(B) and *tet*(M), which suggested that in the presence of *tet*(O), the aforementioned multiple resistance is unlikely to be observed. Such heterogeneity in linkages among genes that confer the same phenotypic resistance highlights the importance of incorporating genetic information when investigating the risk factors for the spread of resistance. The epidemiological factors that underlie the persistence of systematic multiple-resistance patterns warrant further investigations with appropriate adjustments for ecological and bacteriological factors.

## Introduction

The proliferation and dissemination of multidrug resistance in human pathogens is a global concern [1]. Recent intensive studies have begun to uncover the complex mechanisms underlying the resistance patterns. Transmission and persistence of antimicrobial resistance might be attributed to a variety of bacteriological, ecological, and anthropogenic factors [2, 3]. These factors include horizontal transfer of resistance genes among bacteria, the selection pressure exerted by naturally produced antibiotics in the environment, the misuse or overuse of antibiotics in humans and animals, and environmental contamination through livestock slurry and plant wastewater; however, the relative importance of each factor remains to be understood accurately [4].

Enterococci are commensal bacteria that are widely distributed in the intestine of mammals and birds and serve as important nosocomial pathogens [5]. Although enterococci originating from animals are unlikely to cause human infection [6], the transfer of resistance determinants from animal strains to human strains has been reported [7]. Enterococci are recognized to be capable of acquiring and transferring antimicrobial resistance determinants by means of miscellaneous mobile genetic elements from and to other harmful human pathogens [8, 9]. This might occur in animals, the environment, food, or the intestinal tract of people who consume raw or poorly cooked contaminated food including meat [3, 10]. Aslam et al. (2012) reported that enterococci from poultry meat are more likely to harbor clinically important resistance genes as compared with those from pork or beef [11]. This finding raises a major public health concern in Japan, where the practice of consuming raw or undercooked poultry meat is relatively common. However, the contribution of enterococci isolated from meat to the dissemination of resistance determinants remains to be evaluated because of the complex epidemiology of circulating resistant bacteria. At the outset of such studies, data on the prevalence of resistance among enterococci in products like meat is required. Numerous studies have investigated the prevalence and characteristics of antimicrobial resistance among enterococci in poultry products in Japan; however, such studies are typically limited to vancomycin-resistant enterococci and/or poultry products originating from a limited geographical area, and the results obtained often cannot be readily extrapolated [12, 13]. Therefore, in this study, our primary objective was to determine the overall characteristics of antibiotic resistance and resistance determinants prevalent in enterococci isolated from poultry products collected throughout Japan.

Our second objective was to uncover the interrelationships between resistance genes and their effects on the phenotypic expression of resistance. Horizontal gene transfer often involves the spread of multiple resistance genes that are genetically linked to each other [10]. Under the pressure of specific antibiotics and/or heavy metals, various resistance genes that are unrelated to existing antibiotics and/or heavy metals are presumably co-selected, which results in the persistence and spread of multidrug-resistant bacteria [14]. However, limited information has been obtained regarding which patterns of multiple antimicrobial resistance appear systematically. A previous study attempted to identify patterns of gene appearance by examining the unconditional statistical associations between any pair of genes [11]. Furthermore, Ludwig et al. (2013) used an additive Bayesian network (ABN) approach to outline potential interactions between antimicrobial resistance phenotypes [15]. This multivariate approach can be particularly useful for elucidating complex co-appearance of antimicrobial resistance, where the numbers of resistances associate with each other. A single resistance phenotype can be also conferred by several genes, and each gene might be genetically linked in a distinct manner with other genes [16]. These findings collectively suggest that associations between resistances must be understood at the genetic level, in addition to being understood at the phenotypic level. Thus, we applied an ABN modeling approach to the collected data on *Enterococcus faecalis* isolates in order to describe the complex interrelationships between resistances.

## Materials and Methods

### Sampling and isolation of enterococci

**Fig 1:**
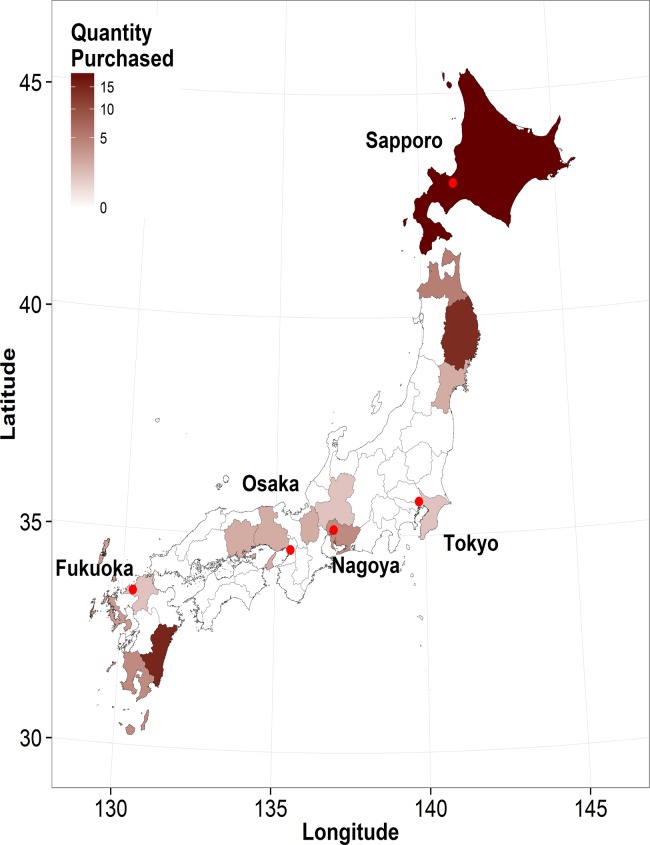
Map of Japan showing the study area. A choropleth map of 46 Japanese administrative areas (prefectures) showing the origin of the purchased domestic chicken products and the quantity of products supplied from each prefecture. The origin of 82 domestic products (52.6%) was unavailable and is not shown. The superimposed points show the locations of the 5 Japanese cities where chicken products were purchased between July and August 2012.

Retail chicken meat and offal products were purchased from 49 large-scale supermarkets in 5 Japanese cities in Tokyo, Hokkaido, Aichi, Osaka, and Fukuoka prefectures between July and August 2012; 9 or 10 chain stores under different brands were purposively selected from Adachi Special Ward (Tokyo: 35.778°N, 139.800°E), Sapporo city (Hokkaido: 42.996°N, 141.261°E), Nagoya city (Aichi: 35.140°N, 136.933°E), Osaka city (Osaka: 34.669°N, 135.502°E), and Fukuoka city (Fukuoka: 33.567°N, 130.355°E) (Fig. 1). These cities are all centers of metropolitan regions in Japan, each with a population of >1 million people, and in these cities, food products are supplied from wide surrounding geographical areas in order to meet the demand of their large populations. Therefore, our sampling approach ensured a reasonable representation that considered both geographical and quantitative consumption of poultry products in Japan. In total, 102 samples of domestic retail chicken meat and 54 domestic offal samples (50 livers, 2 gizzards, and 2 hearts) were collected in approximately equal quantities from the 5 cities. Almost all of the purchased products were identified to be fresh based on the information on the package label; however, 3 products had been previously frozen and thawed before being sold. The products were packaged appropriately to avoid any potential contamination and labeled with the names of prefecture of origin where available; otherwise they were simply labeled as a Japanese product. For 74 products (47.4%), we identified the origin prefectures, which were distributed across Japan (Fig. 1); the common prefectures of origin were Hokkaido (n = 19), Miyazaki (n = 15), and Iwate (n = 14). From each package, 25 g of product was collected, and the weighed samples were vigorously homogenized in 100 mL of phosphate-buffered saline (Nissui Pharmaceutical Co., Ltd, Japan); 2 mL of this homogenate was then enriched for 24 h at 37°C in 8 mL of AC broth base (Nissui Pharmaceutical Co., Ltd) containing sodium azide. One loop of the enriched sample was inoculated on an Enterococcosel Agar plate (Nippon Becton, Dickinson and Company, Japan) and incubated for 48 h at 37°C. We selected 2 presumptive enterococci colonies per sample on the basis of colony morphology and color, transferred these to a brain-heart infusion Agar plate (Nippon Becton, Dickinson and Company), and incubated them for 24 h at 37°C. The obtained isolates were analyzed morphologically and biochemically, and their genus and species (*E*. *faecalis*, *Enterococcus faecium*, or other enterococci) were simultaneously confirmed by means of multiplex PCR performed using primers as previously described [17]. PCR-confirmed isolates were stored in LB broth containing 30% glycerol (v/v) at −80°C. In this study, only *E*. *faecalis* and *E*. *faecium* isolates were used in the experiments described next.

### Antimicrobial susceptibility testing

Isolates were tested for antimicrobial susceptibility by using the broth-microdilution method with Frozen Plate (Eiken Chemical Co. Ltd., Tokyo, Japan). If both *E*. *faecalis* and *E*. *faecium* were isolated from the same products, one isolate per species was tested; otherwise, only one isolate per product was tested. The minimum inhibitory concentration (MIC) was determined using the Clinical and Laboratory Standards Institute guidelines [18] for the following 8 antimicrobials: ampicillin (0.12–128 μg/mL), dihydrostreptomycin (0.25–512 μg/mL), oxytetracycline (0.12–64 μg/mL), erythromycin (0.12–128 μg/mL), chloramphenicol (0.25–512 μg/mL), enrofloxacin (0.12–64 μg/mL), vancomycin (0.12–256 μg/mL), and virginiamycin (0.12–128 μg/mL). These antimicrobials were selected primarily because they are the major antimicrobials tested in Japanese Veterinary Antimicrobial Resistance Monitoring systems [19]; tetracycline, penicillins, macrolides, and aminoglycosides are the antimicrobials most frequently administered in Japanese broiler production [20]. *Staphylococcus aureus* ATCC 29213, *E*. *faecalis* ATCC 29212, *Escherichia coli* ATCC 25922, and *Pseudomonas aeruginosa* ATCC 27853 were used for quality control of the susceptibility testing. Antimicrobial susceptibility for ampicillin, chloramphenicol, erythromycin, vancomycin, and virginiamycin were interpreted in terms of epidemiological cut-off values (i.e., as wild-type or non-wild-type) according to the ECOFF breakpoints established by EUCAST (http://mic.eucast.org/Eucast2/); otherwise the criteria employed in previous Japanese studies were used [18]. For simplicity, wild-type and non-wild-type are referred to as susceptible and resistant, respectively, in the remainder of this report. The breakpoints used are shown in Table 1.

**Table 1:** Prevalence of phenotypic resistance for the tested antimicrobials among isolated Enterococci.

Antimicrobial agent	*E*. *faecalis* from domestic products (n = 113)	*E*. *faecium* from domestic products (n = 25)
Ampicillin (8 μg/mL)	0 (0)	1 (4)
Dihydrostreptomycin (128 μg/mL)	57 (50.4)	5 (20)
Oxytetracycline (16 μg/mL)	66 (58.4)	7 (28)
Chloramphenicol (64 μg/mL)	7 (6.2)	0 (0)
Erythromycin (8 μg/mL)	42 (37.2)	11 (44)
Enrofloxacin (4 μg/mL)	3 (2.7)	8 (32)
Vancomycin (8 μg/mL)	1 (0.9)	1 (4)
Virginamycin (64 or 8 μg/mL)^a^	0 (0)	3 (12)

Prevalence of resistance to each tested antimicrobial in *E*. *faecalis* and *E*. *faecium* isolated from retail poultry products collected in 5 major Japanese cities between July and August 2012.

^a^Breakpoints for *E*. *faecalis* and *E*. *faecium* were 64 and 8 μg/mL, respectively.

### DNA isolation and detection of antimicrobial resistance genes

DNA was isolated from each sample by using a commercial DNA extraction kit (ISOPLANT II; Nippon Gene Co., Ltd., Japan) according to the manufacturer’s instructions. A separate PCR was performed using Ex Taq (TaKaRa Co., Ltd, Japan) to detect each of the following resistance genes: for tetracycline, *tet*(M), *tet*(L), and *tet*(O) (primers and the PCR conditions used are described in [21]); for macrolide, *erm*(B), *erm*(A), and *mef* [21]; for aminoglycoside, *aac*(6’)-Ie-*aph*(2”)-Ia, *aph*(3’)-IIIa, and *ant*(6)-Ia [22]; and multidrug resistance gene, *cfr* [23]. Together with a negative control, sequence-confirmed positive controls were used in all cases except for *mef* and *cfr*, which were not available for this study; these genes were included for a preliminary screening because, to our knowledge, limited information is available on their epidemiology in Japan. The obtained nucleotide sequences of the resistance genes have been deposited at DDBJ/EMBL/GenBank under the accession numbers LC016843–LC016850.

### Statistical analysis

All statistical analyses were performed using R version 3.0.2 (R Core Team, 2013; available from http://www.R-project.org/). The chi-squared test was used to examine differences in proportions throughout the analyses. An ABN model was constructed using R package abn.

#### Overview of additive Bayesian network

A Bayesian network (BN) is a joint probability model that describes interdependencies between variables in the form of a directed acyclic graph (DAG). In a BN, the focus is on discovering structure in an objective manner; unlike path analyses, a BN attempts to determine the optimal graphical model directly from empirical data [24]. In graphical statistical modeling, no distinction is made between covariates and response variables. All variables are treated as random variables and potential dependencies between them are allowed to be present. This distinct feature of a BN appears to be suitable for elucidating complex interrelationships in antimicrobial resistance data.

In this study, the ABN model was constructed to identify associations between each antimicrobial resistance gene and selected antimicrobial phenotypes, which were treated as random variables (nodes in DAG). Resistance genes and phenotypes that occurred in <10% of *E*. *faecalis* isolates were excluded from the analysis. The benefit of using the ABN model, rather than the conventional nominal or ordinal response model, is that categorical outcomes can be described as a function of distinct sets of variables [25]. The results of a previous study showed that the degree of resistance varied among resistance genes that conferred the same resistance phenotype [26]. This phenomenon can be investigated using the ABN approach. Therefore, the phenotypic resistances were further categorized as low- and high-resistance, if applicable. Because no universal definition was available for this categorization, we arbitrarily defined high-resistance isolates as isolates whose MICs were beyond the upper boundary used in our experiments. In the ABN model, the direction of arcs cannot be identified without certain prior knowledge [25]. In this study, we prohibited any arcs stemming from the nodes of resistance phenotype for 2 reasons: (1) it is biologically reasonable to consider that resistance is conferred by resistance genes and not vice versa; and (2) model simplicity; models featuring arcs between phenotypes are plausible. However, given the limited sample size in this study, we focused on the effects of the examined genes on the antimicrobial resistance phenotypes that were measured.

#### Structure learning

The maximum number of parents per node was determined as follows. Initially, only one parent per node was allowed, and the most probable DAG was identified using the well-established exact-search method [27] and the log marginal likelihood was obtained. This step was repeated each time, with the maximum number of parents per node being increased by one until the log marginal likelihood of the identified DAG did not improve further. This model served as the initial model, and the marginal posterior density for each parameter was subsequently estimated. Each estimated parameter was then checked, both visually and numerically. Marginal densities obtained from the initial model are shown in S1 Fig.

#### Adjustment for over-fitting

In order to assess over-fitting, we used a parametric bootstrapping approach [28]. Based on the marginal density of each parameter, a bootstrap dataset of the same size as the original observed data (i.e., n = 113) was generated by using the Markov chain Monte Carlo simulation in JAGS software (http://mcmc-jags.sourceforge.net/). The bootstrap simulation was performed 10,000 times, and an exact search was then conducted for each simulated dataset; this generated a total of 10,000 DAGs. The arcs present in the initial model were retained in the final model only if they were recovered in >50% of the bootstrap simulations [29]. The distribution of the number of total arcs in each DAG and the frequency of each arc recovered in the course of the simulations are presented in S1 Table and S2 Fig., respectively. The direction of arcs could not be determined because of likelihood equivalence; therefore, the frequency of appearance for each arc was measured by collapsing over arc direction, and graphical networks are presented without arc direction [25]. S3 Fig. shows the marginal densities obtained from the final model. The data used and the R code for the initial exact-search method are presented in S1 Dataset and S1 Text, respectively.

## Results

### Prevalence of resistance phenotypes and genes

In this study, we sampled 156 domestic retail poultry products; *E*. *faecalis* and *E*. *faecium* were isolated from 113 (72.4%) and 25 (16.0%) products, respectively. Table 1 shows the prevalence of each antimicrobial-resistant strain of *E*. *faecalis* and *E*. *faecium* isolated from the poultry products. One isolate of *E*. *faecalis* and one of *E*. *faecium* were resistant to vancomycin according to the ECOFF; however, both their MICs were 8 μg/mL, which suggested that the level of resistance was very low. Table 2 shows the prevalence of each antimicrobial resistance gene that was analyzed in this study. Only *E*. *faecalis* isolates were found to harbor *aac*(6’)-Ie-*aph*(2”)-Ia. The prevalence of resistance determinants among the *E*. *faecium* isolated was lower than that among *E*. *faecalis* isolated from the same samples, although we did not analyze this difference statistically. *erm*(A), *mef* and *cfr* were not detected in any of the samples.

**Table 2:** Prevalence of tested resistance genes among isolated Enterococci.

Antimicrobial resistance gene	*E*. *faecalis* from domestic products (n = 113)	*E*. *faecium* from domestic products (n = 25)
*aac*(6')-Ie-*aph*(2")-Ia	5 (4.4)	0 (0)
*aph*(3')-IIIa	28 (24.8)	1 (4)
*ant*(6)-Ia	23 (20.4)	1 (4)
*tet*(L)	52 (46.0)	6 (24)
*tet*(M)	38 (33.6)	5 (20)
*tet*(O)	15 (13.3)	0 (0)
*erm*(A)	0 (0)	0 (0)
*erm*(B)	32 (28.3)	2 (8)
*mef*	0 (0)	0 (0)
*cfr*	0 (0)	0 (0)

Prevalence of antimicrobial resistance genes tested among *E*. *faecalis* and *E*. *faecium* isolated from retail poultry products collected in 5 major Japanese cities between July and August 2012.

### Additive Bayesian network application to resistance phenotypes and genes

**Fig 2:**
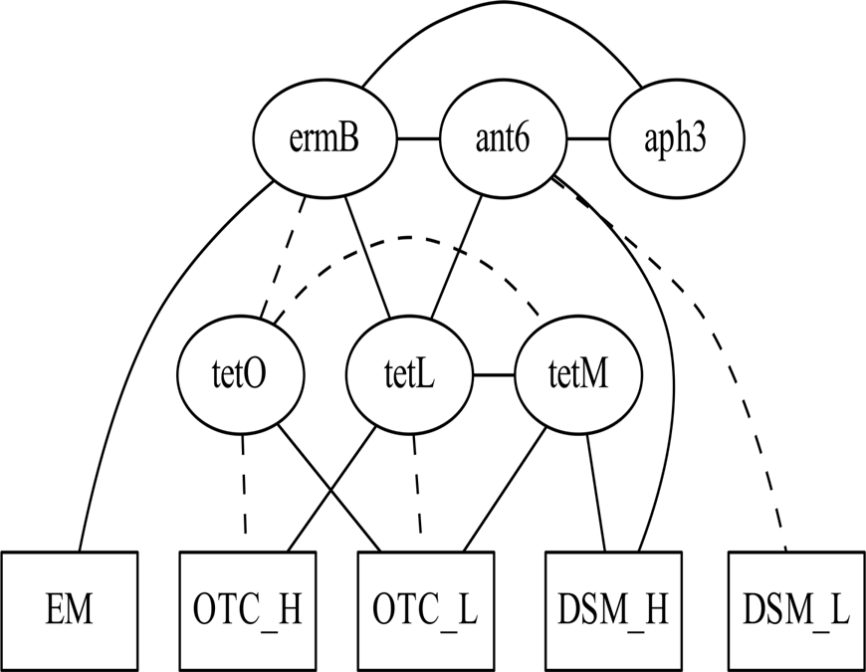
Initial optimal additive Bayesian network model. Identified interrelationships between selected antimicrobial resistance genes and their effects on the phenotypic expressions of resistance among 113 isolates of *Enterococcus faecalis* from domestic poultry products collected from retail shops in 5 major Japanese cities between July and August 2012. Abbreviations: ant6: *ant*(6)-Ia; aph3: *aph*(3’)-IIIa; EM: erythromycin resistance; DSM_L: MIC for dihydrostreptomycin ≥128 μg/mL and ≤512 μg/mL; DSM_H: MIC for dihydrostreptomycin >512 μg/mL; OTC_L: MIC for oxytetracycline ≥16 μg/mL and ≤64 μg/mL; OTC_H: MIC for oxytetracycline >64 μg/mL. Solid lines and dashed lines represent positive and negative associations between variables, respectively.

**Fig 3:**
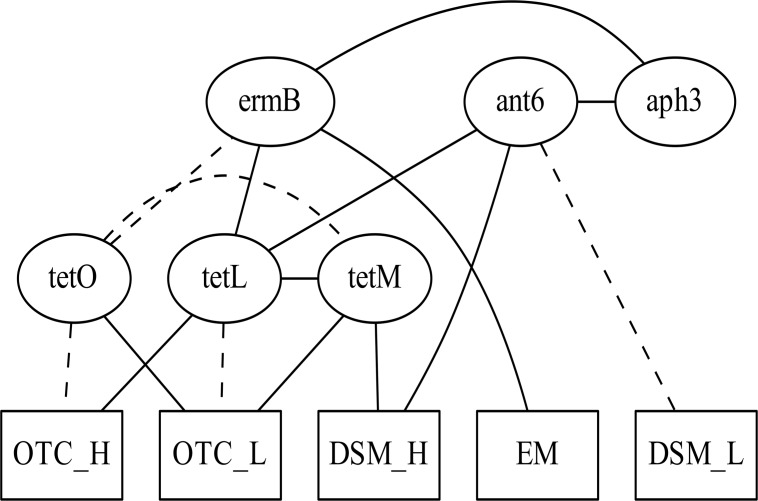
Final globally optimal additive Bayesian network model after adjustment for over-fitting. Final additive Bayesian network model after removing arcs that appeared in <50% of bootstrappings for the interrelationships between selected antimicrobial resistance genes and phenotypes. Solid lines and dashed lines represent positive and negative associations between variables, respectively. Fig. 2 lists the variable names.

Next, an ABN model was constructed to analyze the interrelationships between resistance genes and phenotypes in *E*. *faecalis*. We excluded these resistance genes that were detected in <10% of *E*. *faecalis* isolates: *aac*(6’)-Ie-*aph*(2”)-Ia, *erm*(A), *mef*, and *cfr*. The resistance phenotypes for dihydrostreptomycin, erythromycin, and oxytetracycline were only included in the model because other phenotypes occurred in <10% of isolates. Each resistance phenotype for dihydrostreptomycin and oxytetracycline was further categorized as high (dihydrostreptomycin, >512 μg/mL; oxytetracycline, >64 μg/mL) or otherwise low. Erythromycin resistance could not be categorized, because the MICs of very few isolates were between the upper boundary (128 μg/mL) and the breakpoint (8 μg/mL). Therefore, 5 nodes representing resistance phenotypes were included in the model. We determined that a maximum of 3 parents per node was sufficient to maximize the fit of the model. Subsequently, we performed an exact search—with the upper limit set at 3 parent nodes for each node—in order to identify the tentative associations between genes and phenotypes (Fig. 2). After adjustment for over-fitting, the association between *ant*(6)-Ia and *erm*(B) was not robust and was therefore excluded (Fig. 3). This removed unstable marginal densities related to the node *ant*(6)-Ia (S1 and S3 Figs). Table 3 shows the posterior marginal log odds for each arc. The presence of *tet*(L) was positively associated with high-level oxytetracycline resistance (>64 μg/mL), whereas the presence of *tet*(O) and *tet*(M) was positively associated with low-level oxytetracycline resistance. These analyses revealed complex interrelationships between genes, as discussed in the next section.

**Table 3:** Posterior marginal log odds ratios for parameters.

Arc		Log odds	
Child	Parent	Median	Credibility interval	
		50%	2.50%	97.50%
*aph*(3’)-IIIa	*ant*(6)-Ia	17.31	6.43	43.35
*aph*(3’)-IIIa	*erm*(B)	15.00	4.39	40.95
*erm*(B)	*tet*(O)	−2.90	−6.23	−0.98
*erm*(B)	*tet*(L)	2.30	1.34	3.38
*tet*(M)	*tet*(O)	−3.21	−6.56	−1.29
*tet*(M)	*tet*(L)	2.17	1.27	3.15
*tet*(L)	*ant*(6)-Ia	2.18	1.08	3.52
Dihydrostreptomycin (Low)^a^	*ant*(6)-Ia	−2.58	−5.90	−0.78
Dihydrostreptomycin (High)^b^	*ant*(6)-Ia	7.53	4.82	12.13
Dihydrostreptomycin (High)^b^	*tet*(M)	3.33	1.36	6.63
Erythromycin	*erm*(B)	24.88	5.45	70.54
Oxytetracycline (Low)^c^	*tet*(M)	3.27	1.73	5.12
Oxytetracycline (Low)^c^	*tet*(O)	6.03	3.81	8.78
Oxytetracycline (Low)^c^	*tet*(L)	−3.93	−6.28	−2.16
Oxytetracycline (High)^d^	*tet*(O)	−4.25	−6.66	−2.37
Oxytetracycline (High)^d^	*tet*(L)	7.10	4.97	10.51

Posterior estimates for remaining arcs obtained from the final ABN model after bootstrapping. The median and 95% credibility intervals for each parameter estimate are shown.

^a^MIC for dihydrostreptomycin: ≥128 μg/mL and ≤512 μg/mL.

^b^MIC for dihydrostreptomycin: >512 μg/mL.

^c^MIC for oxytetracycline: ≥16 μg/mL and ≤64 μg/mL.

^d^MIC for oxytetracycline: >64 μg/mL.

## Discussion

The concomitant spread of multiple resistance genes through mobile genetic elements such as transposons, integrons, and plasmids is now a well-recognized problem [30]. This study has highlighted the complex interrelationships between resistance genes and their phenotypic expressions. In particular, we confirmed that each *tet* gene is likely to exhibit distinct linkage patterns with other resistance genes. This finding has a major implication for epidemiological studies that examine risk factors associated with the presence and transmission of antimicrobial resistance; the sets of risk factors associated with a specific resistant phenotype might vary depending on which resistance genes exist and are responsible for the resistance. Therefore, improving our understanding of systematic patterns of multiple antimicrobial resistances is a crucial step toward accurately identifying the underlying factors associated with the emergence and spread of bacteria that exhibit multiple resistances [15]. Together with using a meaningful resistance pattern as an outcome, epidemiological studies must appropriately adjust for the presence of numerous confounders, including ecological factors and the chronology of antimicrobial administrations, as highlighted in a recent review [4].

The presence of *ant*(6)-Ia and *erm*(B) was associated with high resistance to dihydrostreptomycin and erythromycin, respectively [31, 32]. By contrast, among the low-level dihydrostreptomycin-resistant isolates (n = 28), only one isolate was positive for *ant*(6)-Ia, which could be explained by the finding that the aminoglycoside resistance of enterococci is intrinsically of a low to moderately level [31]. The presence of each *tet* gene was found to affect oxytetracycline resistance distinctly. Whereas high-level oxytetracycline resistance (>64 μg/mL) was positively associated only with the presence of *tet*(L), low resistance was positively associated with the presence of *tet*(O) and *tet*(M). A linkage between *tet*(L) and *tet*(M) was suggested, which agrees with previous findings [26, 33]. Schwaiger et al. (2009) reported that the coexistence of these 2 genes enhances the MICs against doxycycline [26]. In future studies, this could be investigated by using our current approach and testing a broader range of MICs of tetracycline. A negative association was identified between the presence of *tet*(O) and *tet*(M), both of which confer tetracycline resistance through ribosomal protection [34]. Blake et al. (2003) suggested that *E*. *coli* benefit little by concurrently carrying 2 different genes that confer tetracycline resistance through efflux pump systems [16]. This might explain the identified negative associations between *tet*(O) and *tet*(M), but the association between resistance genes with similar roles must be researched further.

Multiple resistances against streptomycin, kanamycin, erythromycin, and tetracycline occur frequently among animal-origin enterococci [35]. Our results identified linkages between *erm*(B) and *tet*(L), *tet*(L) and *ant*(6)-Ia, *ant*(6)-Ia and *aph*(3’)-IIIa, and *aph*(3’)-IIIa and *erm*(B), corroborating that this multiple resistance profile is systematic rather than random. In accord with our results, previous studies have reported a genetic linkage between *tet*(L) and *erm*(B) [36, 37], and Werner et al. (2001) suggested the presence of a gene cluster containing *ant*(6)-Ia and *aph*(3’)-IIIa [38]. The associations between *tet*(M), *erm*(B), and *aph*(3’)-IIIa through Tn*1545*-like transposons have been also extensively observed [39]. However, in this study, we did not observe an association between *tet*(M) and *erm*(B). De Leener et al. (2004) reported that Tn*1545*-like transposons appeared frequently among enterococci isolated from humans and pigs [40], but this transposon was found to occur comparatively less among isolates from broilers [41]. These findings might explain why we did not observe a linkage between *erm*(B) and *tet*(M). Furthermore, we identified a negative association between *tet*(O) and *erm*(B). Although we do not have a clear explanation for this negative association, the colocalization of these 2 genes is presumably uncommon [42]. Interestingly, our final model showed an association between the presence of *tet*(M) and high-level resistance against dihydrostreptomycin. This might imply a potential relationship between *tet*(M) and other unexamined genes such as *ant*(3”)-Ia that are responsible for streptomycin resistance; however, further molecular studies are required to identify the mechanism underlying this association.

We showed that each *tet* gene exhibits a distinct linkage pattern to other genes. The diversity in the linkage to other resistance genes among distinct *tet* genes has also been described in *E*. *coli* [16]. Notably, in this study, the presence of *erm*(B) was positively associated with the presence of *tet*(L) and negatively associated with the presence of *tet*(O). This finding can potentially generate 2 very different scenarios if these associations were inherently preserved. For instance, the use of erythromycin might or might not co-select tetracycline resistance depending on the type of *tet* genes present. If tetracycline resistance is treated as a single binary variable under the presence of these distinct mechanisms, the deduced effect of risk factors might be biased. Although this concern was raised almost a decade ago [43], the adjustment necessary does not appear to be fully employed in epidemiological studies. Such heterogeneity might also apply to other resistance genes, and this must be carefully considered in future epidemiological studies.

We detected a low prevalence of *aac*(6’)-Ie-*aph*(2”)-Ia in this study. Watanabe et al. (2009) reported high prevalence of this gene among enterococci isolated from humans in Japan [22]. Although the geographical location and time between their study and this study cannot be compared, this discrepancy potentially suggests that the enterococcus populations circulating among humans and retail chicken meat might differ. Although *aac*(6’)-Ie-*aph*(2”)-Ia was identified in only 4.4% (n = 5) of the isolates, its genetic profile was intriguing: *aac*(6’)-Ie-*aph*(2”)-Ia did not appear together with *aph*(3’)-IIIa, *ant*(6)-Ia, *erm*(B), or *tet*(O), but all isolates that were positive for *aac*(6’)-Ie-*aph*(2”)-Ia were also positive for *tet*(L). High-level gentamycin resistance, which is conferred by *aac*(6’)-Ie-*aph*(2”)-Ia, limits therapeutic options for enterococcus infections in humans [5]; therefore, the linkage of gentamycin resistance to other resistance genes must be further examined.

Here, we have described the prevalence of both antimicrobial resistance phenotypes and genes among *E*. *faecalis* isolated from domestic poultry products in Japan. Because massive amounts of poultry products are widely circulated beyond regional and national boundaries, future studies must elucidate whether bacteria isolated from imported products exhibit distinct resistance characteristics. We observed a certain level of heterogeneity when we conducted a preliminary survey on 15 *E*. *faecalis* isolates from 20 imported poultry products that were purchased in the same retail stores as were the domestic products used in this study; the prevalence of resistance against dihydrostreptomycin, erythromycin, and enrofloxacin was 80.0%, 73.3%, and 13.3%, respectively, and these levels were significantly higher than those in domestic products (*P* = 0.03, 0.008, and 0.04, respectively). Similarly, the resistance genes *ant*(6)-Ia and *erm*(B) were detected in 46.7% and 73.3% of the isolates, respectively, both significantly higher than the levels in domestic-product isolates. We also detected *erm*(A) in one isolate from imported products. Additional studies are required to understand whether or not patterns of multiple antimicrobial resistance differ between bacteria isolated from domestic and imported products.

This study has certain limitations. First, the identified statistical associations between genes must be interpreted with caution, because these findings do not address the physical linkages between genes, such as colocalization on same mobile genetic elements. Molecular studies are required to comprehensively confirm the hypothetical linkages suggested here. Another limitation of this study is that we arbitrarily defined the threshold separating low- and high-level resistance. This could not be avoided because no quantitative information is available regarding how resistance determinants affect MICs. The use of alternative thresholds could alter the arcs from gene nodes to phenotype nodes, but it would not substantially affect the identified interrelationships between genes. We also examined a limited number of resistance phenotypes and genes in a small number of isolates; conducting a similar study on a larger scale would provide valuable information on the systematic patterns identified for multiple antimicrobial resistances. In this study, we did not consider the geographical origins of chicken because they could not be identified for the majority of the products. Although we did not observe a significant difference in the prevalence of each resistance phenotype between the 5 cities (chi-squared test; *P* > 0.1), detailed information on product source must be included in future studies in order to account for any potential spatial autocorrelations in antimicrobial resistance patterns [43].

Despite the aforementioned limitations, we believe that this study highlights the complex interrelationships between resistance determinants and emphasizes the multifaceted characteristics of resistance-phenotype patterns. The monitoring of phenotypic profiles of resistance, which is typically conducted under current surveillance systems, might limit our understandings of how resistance spreads [44]. Therefore, an improved understanding of the epidemiology of resistance genes is required together with an understanding of resistance phenotypes; however, obtaining genetic profiles on large numbers of isolates is expensive and time-consuming. Our study suggests that the strength of resistance might be associated with the presence of specific genes. If the measured MIC values indicate the presence of specific resistance genes, phenotypic profiles could serve as a proxy for genetic profiles. This might reduce the aforementioned misclassifications in risk-factor studies without the requirement for detecting each resistance determinant. To minimize the spread of antimicrobial resistance, additional studies must be conducted to further understand the genetic epidemiology behind phenotypic resistance and multiple resistances.

## Conclusion

This study determined the prevalence of phenotypes and genes responsible for antimicrobial resistance among *E*. *faecalis* isolated from retail poultry products in Japan. Additive Bayesian network modeling was used to uncover complex interrelationships between resistance genes. The analyses revealed heterogeneity in the linkage patterns among the 3 *tet* genes examined. When investigating the risk factors for the appearance of antimicrobial resistance, ignoring the various complex relationships between resistance genes might lead to a failure to elucidate the causal effects, if any, of the factors examined. An improved understanding of multiple antimicrobial-resistance patterns at the genetic level is required, and such complex genetic information must be fully considered in future epidemiological studies in order to minimize any potential misclassifications of outcomes.

## Supporting Information

S1 Dataset Data used for ABN. (113 observations for 11 variables)
(CSV)

S1 Fig Marginal densities obtained from the initial DAG. (PDF)

S2 Fig Distribution of the total number of arcs that appeared in DAG over 10,000 simulations. (PDF)

S3 Fig Marginal densities obtained from the final DAG. (PDF)

S1 Table Frequency of each arc recovered over 10,000 simulations. (DOCX)

S1 Text R code for initial ABN model. (DOCX)
