# Supplementary figures and images for: Unraveling Antimicrobial Resistance Genes and Phenotype Patterns among Enterococcus faecalis Isolated from Retail Chicken Products in Japan
Source: PLoS One. 2015 Mar 17;10(3):e0121189. doi: 10.1371/journal.pone.0121189 (PMC4363150; doi:10.1371/journal.pone.0121189)

**Frequency out of 10000**

2000

1000

0

8

12

16

20

**Number of total arcs appeared in each bootstrap**

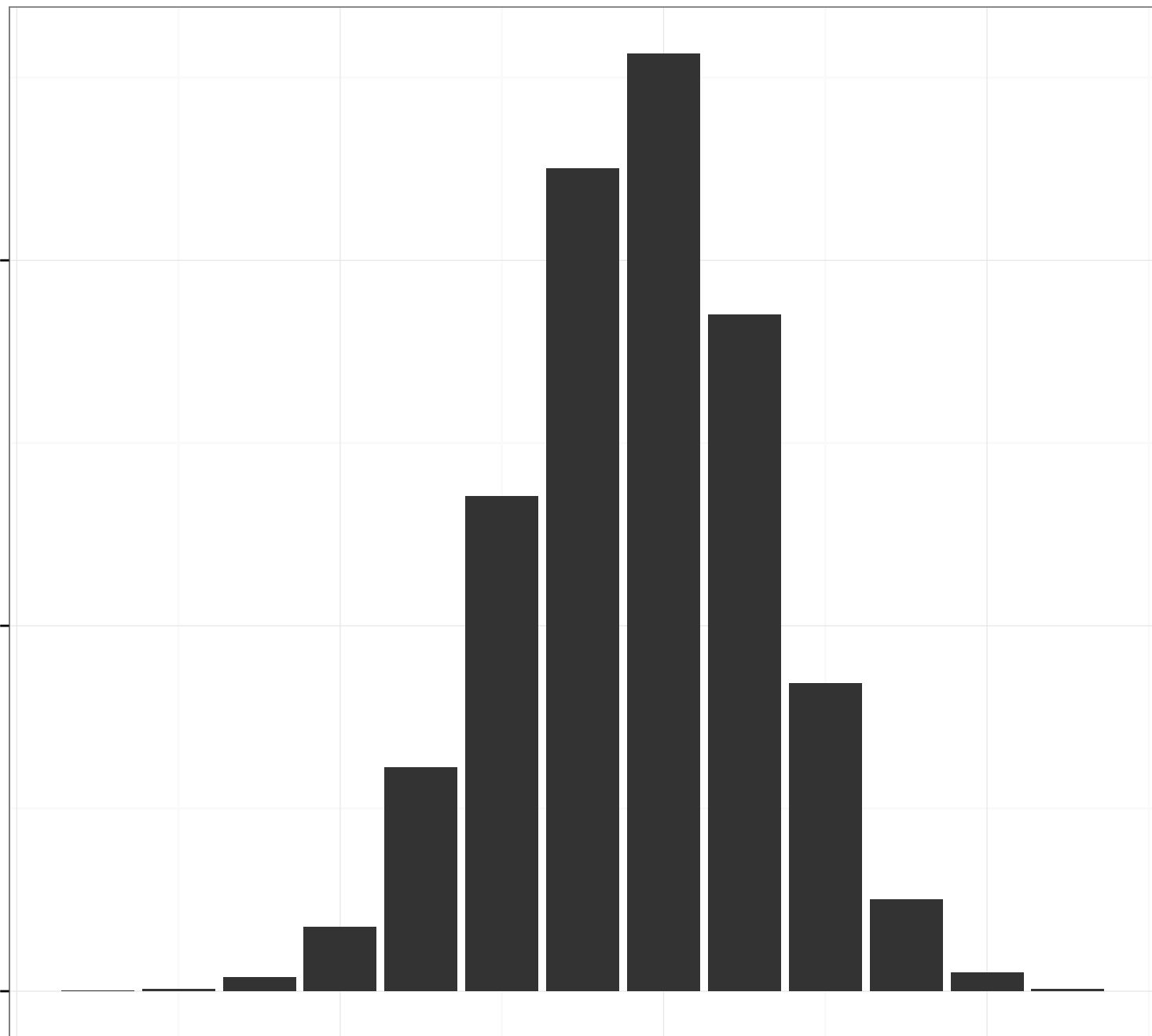

Supplement: S2 Fig — (PDF) [file pone.0121189.s003.pdf]
