# Supplementary material for: Unraveling Antimicrobial Resistance Genes and Phenotype Patterns among Enterococcus faecalis Isolated from Retail Chicken Products in Japan
Source: PLoS One. 2015 Mar 17;10(3):e0121189. doi: 10.1371/journal.pone.0121189 (PMC4363150; doi:10.1371/journal.pone.0121189)
Supplement: S3 Fig — (PDF) [file pone.0121189.s004.pdf]

**ant6 : ant6|(Intercept)**

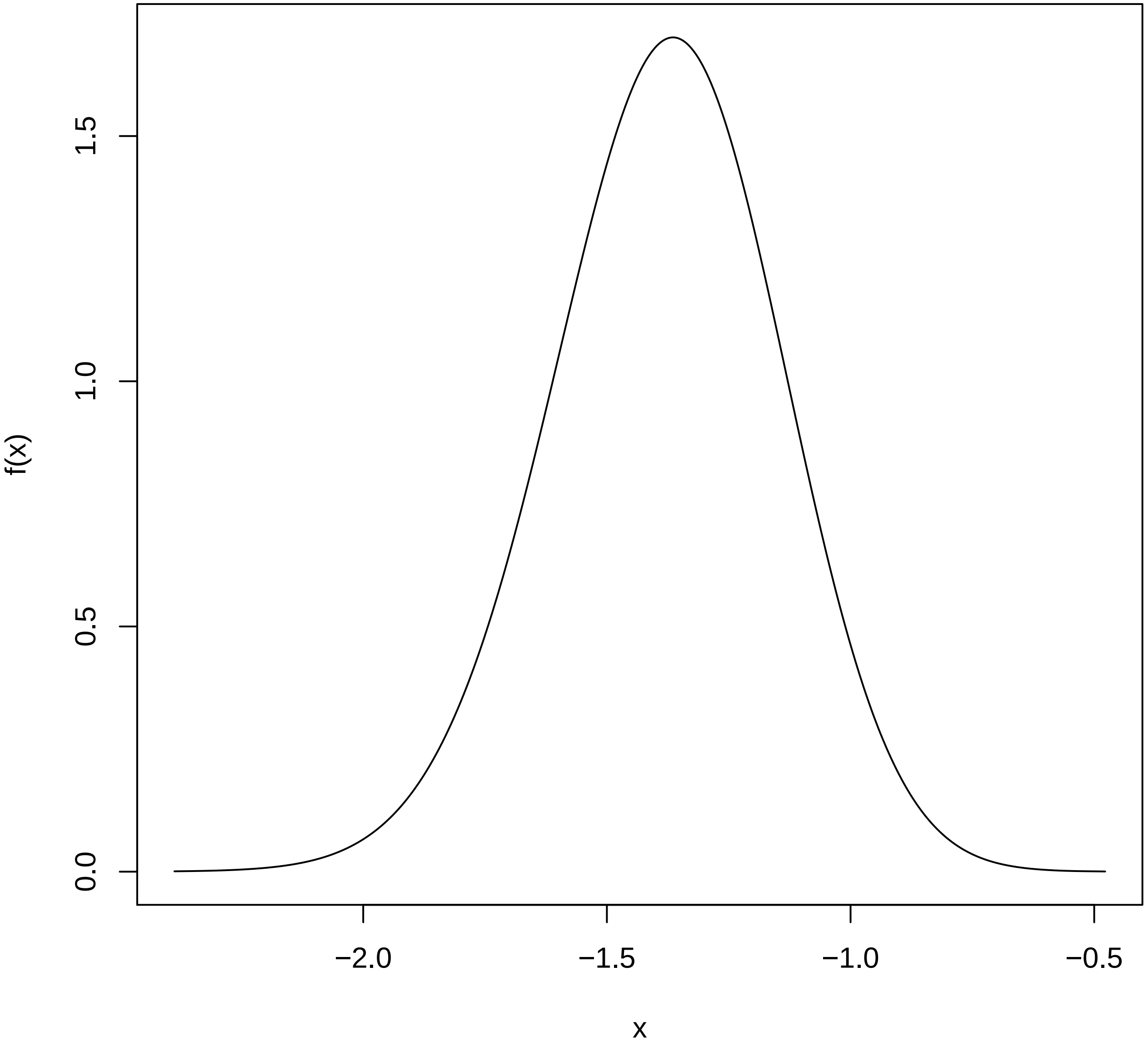

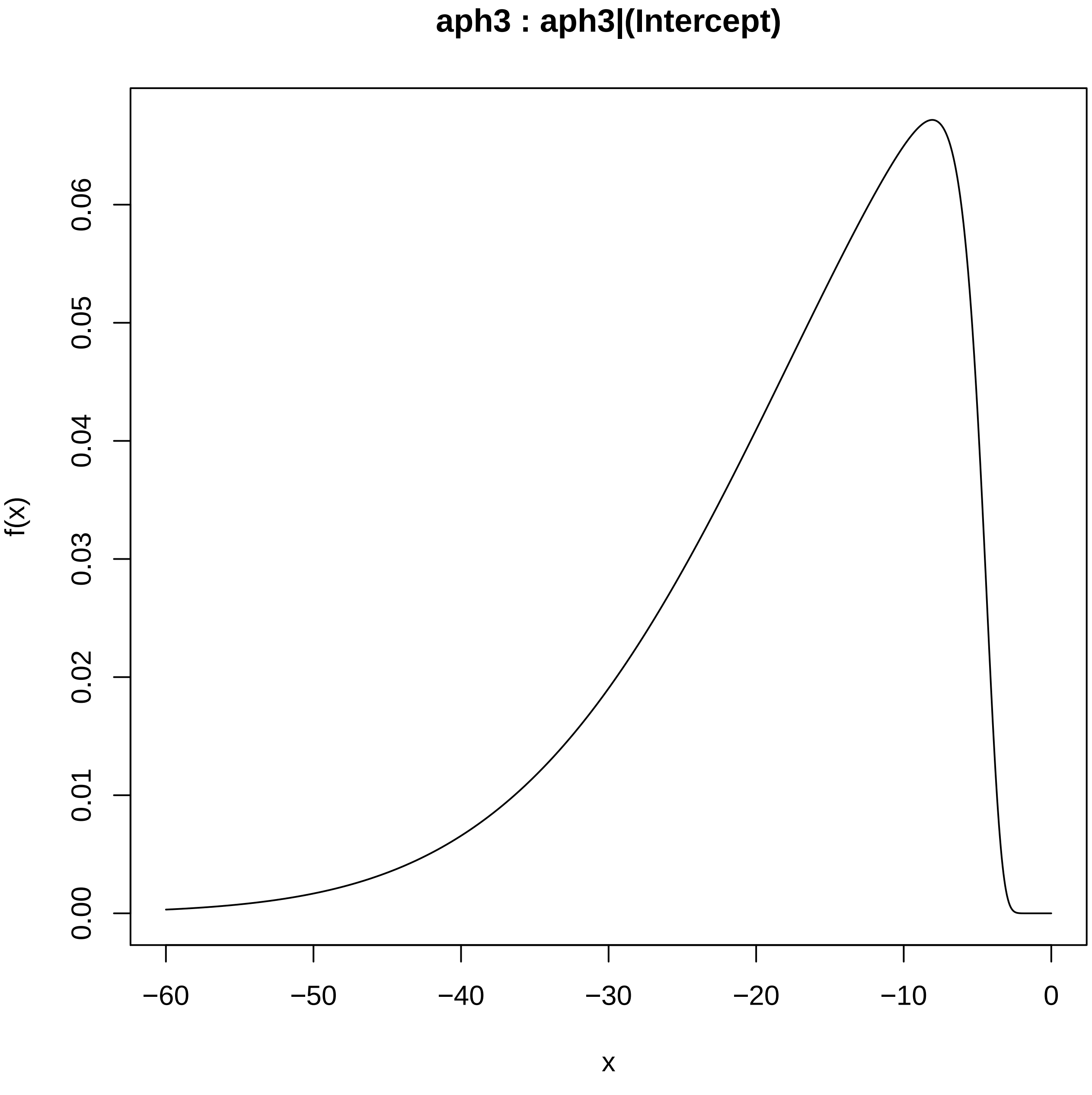
aph3 : aph3|(Intercept)
f(x)
0.00
0.01
0.02
0.03
0.04
0.05
0.06
−60
−50
−40
−30
−20
−10
0
x

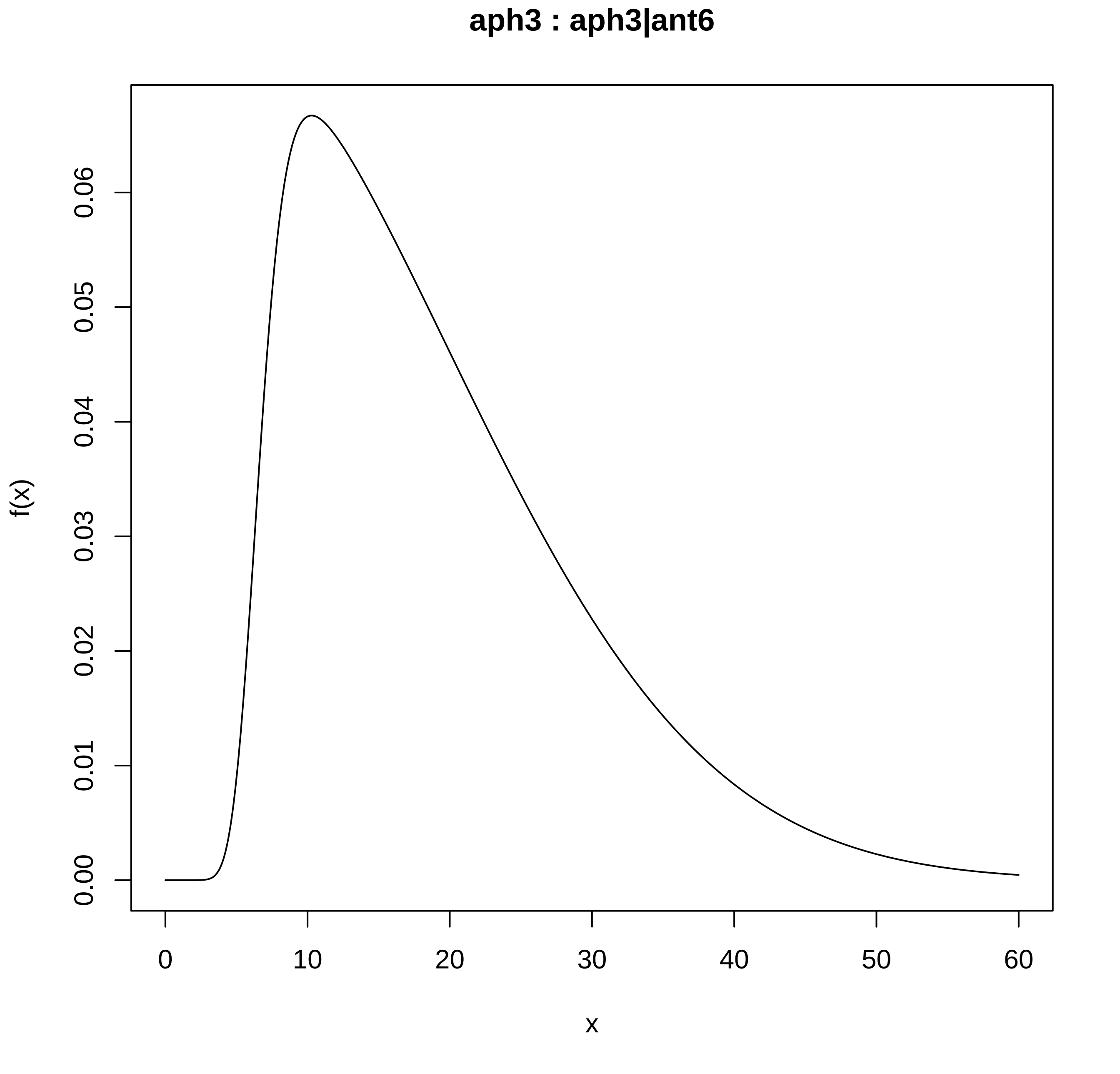
aph3 : aph3|ant6
f(x)
0.00
0.01
0.02
0.03
0.04
0.05
0.06
0
10
20
30
40
50
60
x

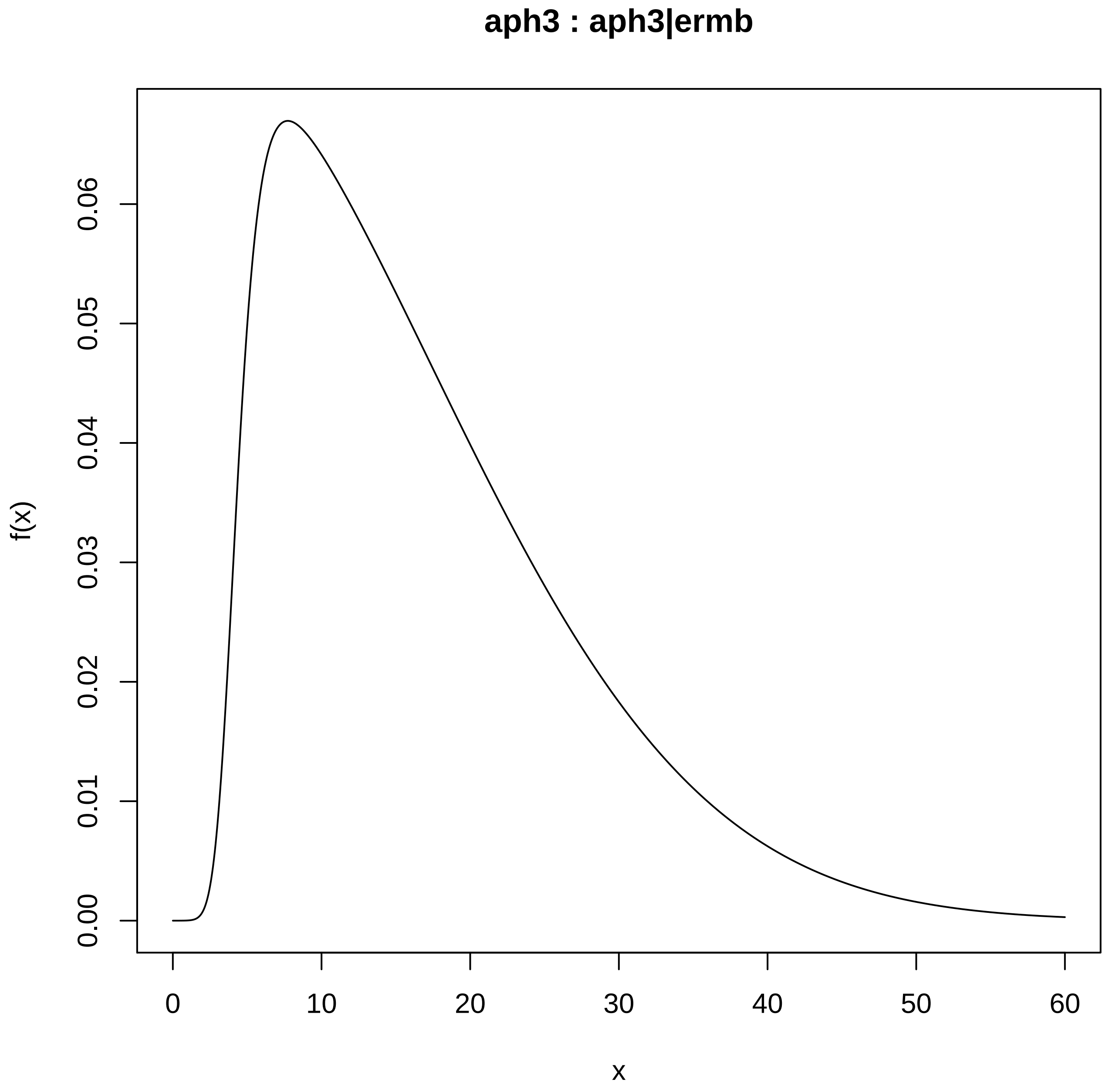
aph3 : aph3|ermb
f(x)
0.00
0.01
0.02
0.03
0.04
0.05
0.06
0
10
20
30
40
50
60
x

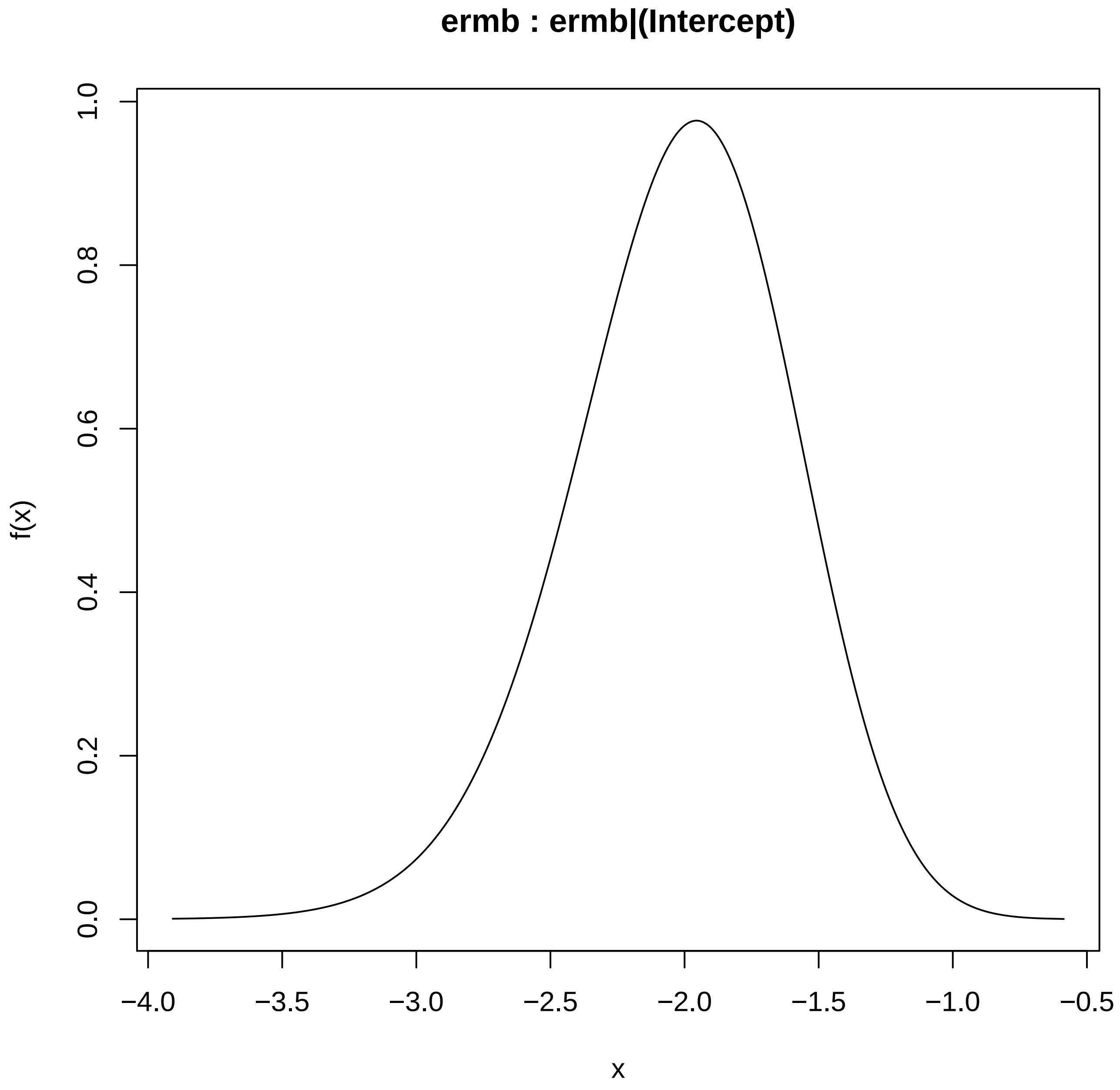

ermb : ermb|(Intercept)
f(x)
0.0
0.2
0.4
0.6
0.8
1.0
−4.0
−3.5
−3.0
−2.5
−2.0
−1.5
−1.0
−0.5
x

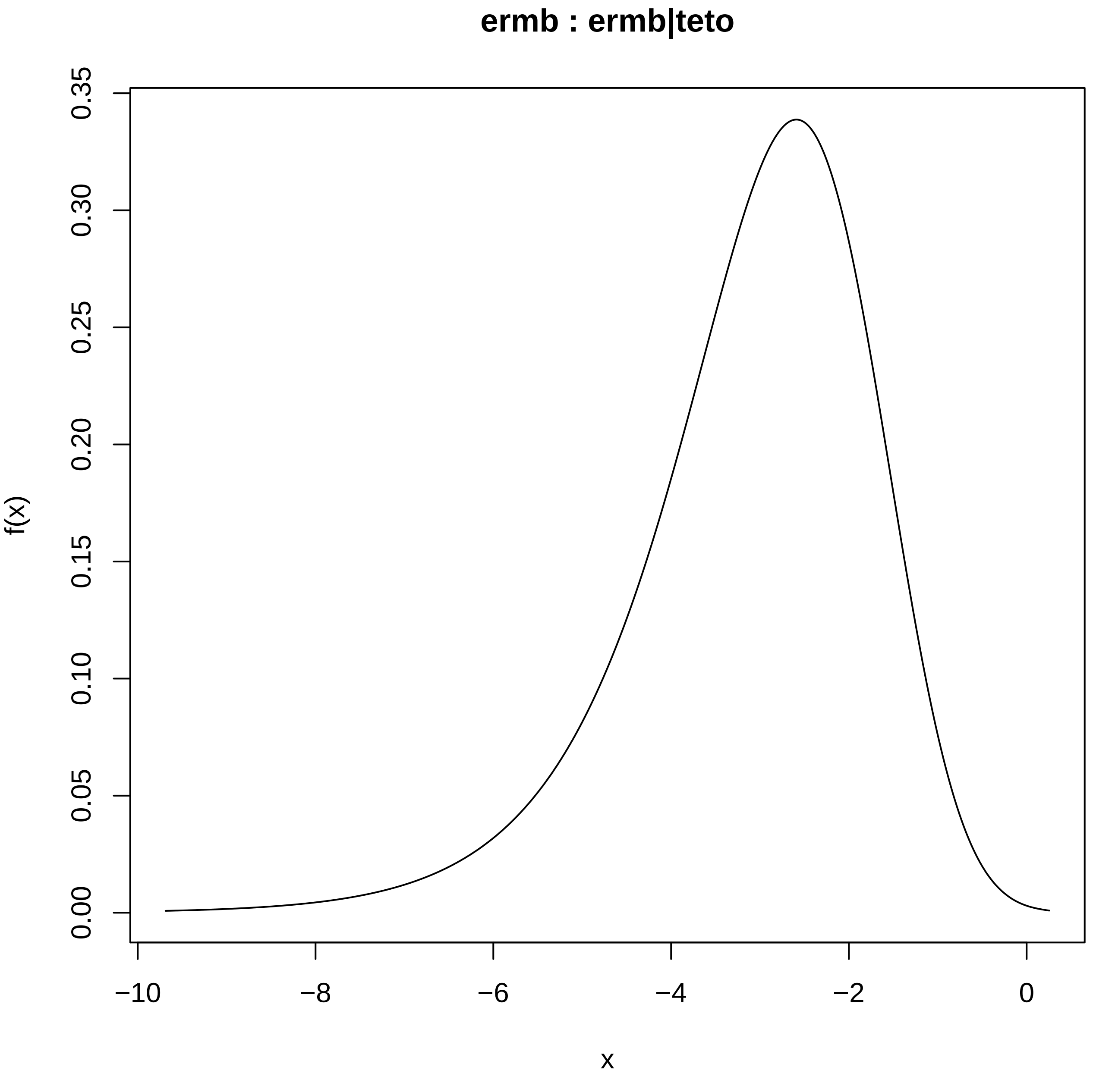
ermb : ermb|teto
0.00
0.05
0.10
0.15
0.20
0.25
0.30
0.35
f(x)
−10
−8
−6
−4
−2
0
x

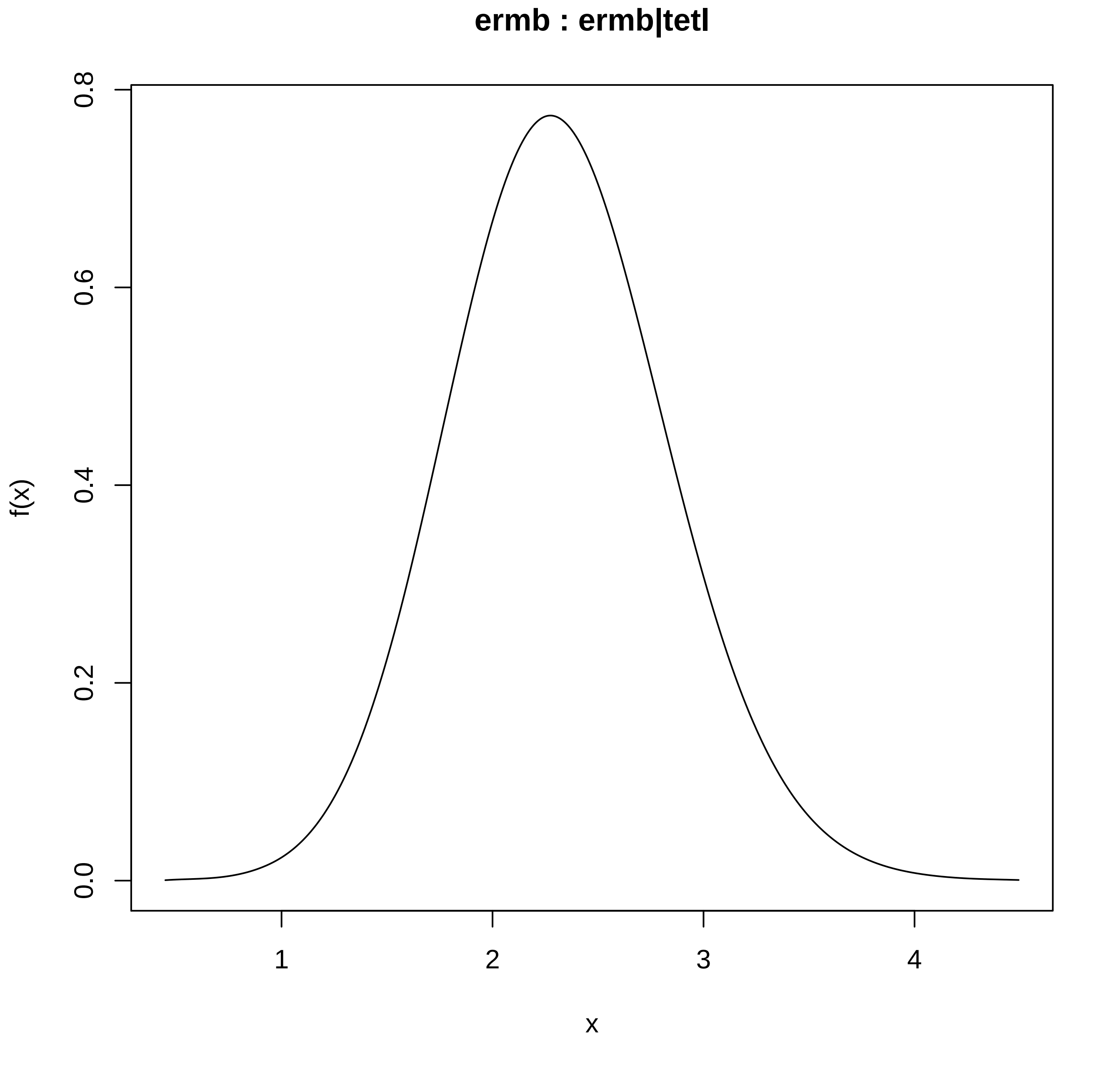
ermb : ermb|tetl
f(x)
0.0
0.2
0.4
0.6
0.8
1
2
3
4
x

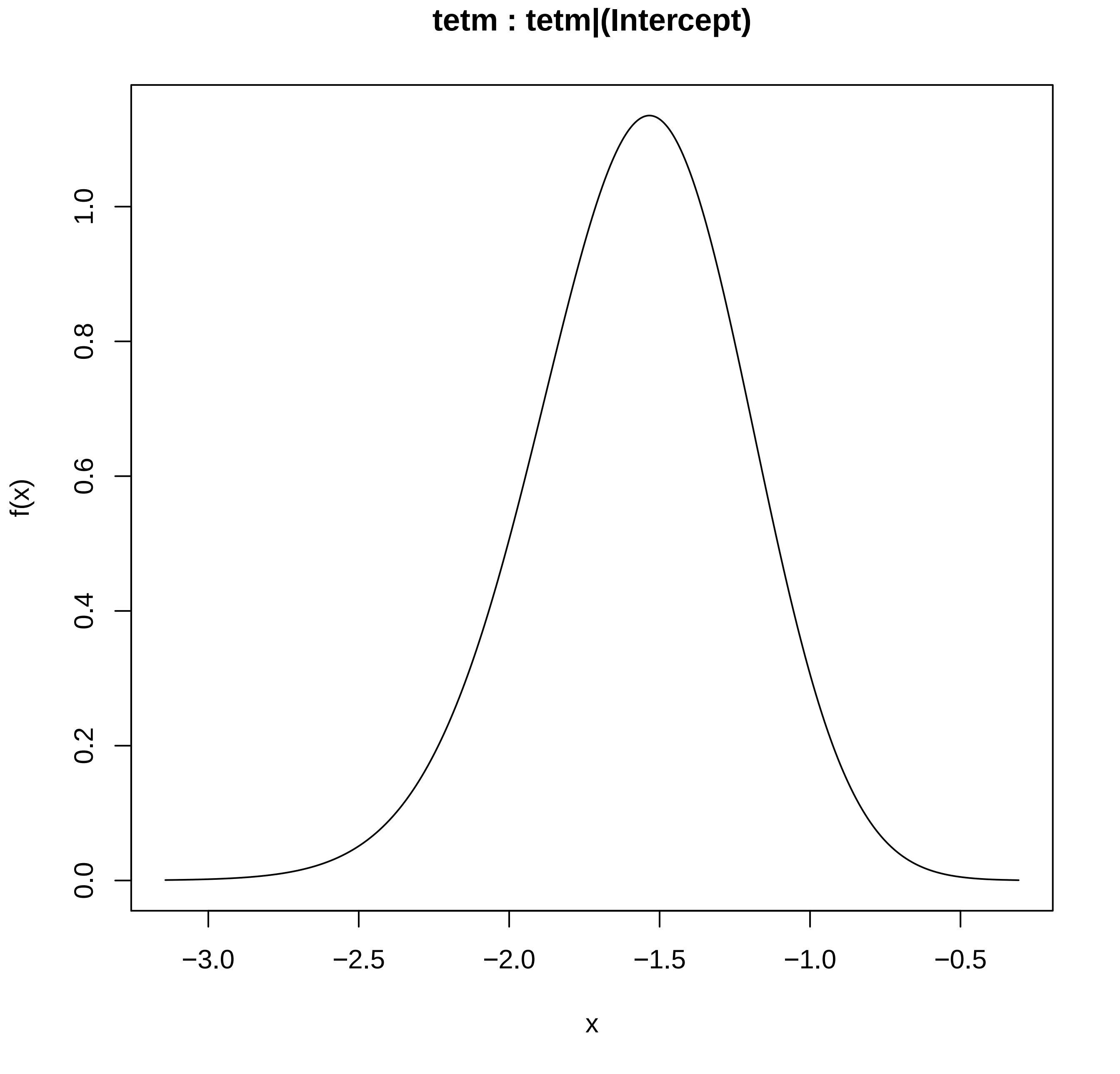
tetm : tetm|(Intercept)
f(x)
1.0
0.8
0.6
0.4
0.2
0.0
−3.0
−2.5
−2.0
−1.5
−1.0
−0.5
x

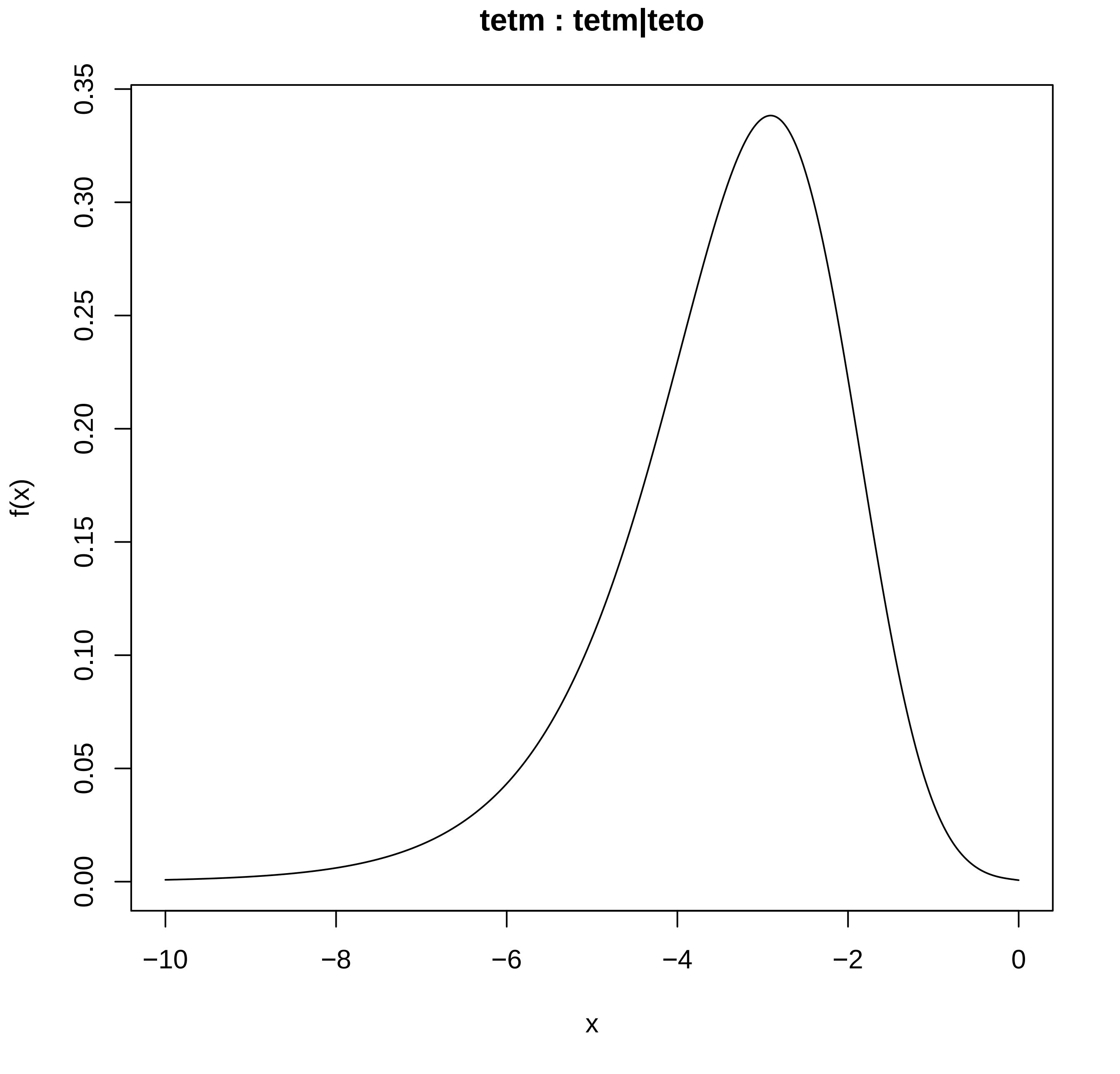

tetm : tetm|teto
0.00
0.05
0.10
0.15
0.20
0.25
0.30
0.35
f(x)
−10
−8
−6
−4
−2
0
x

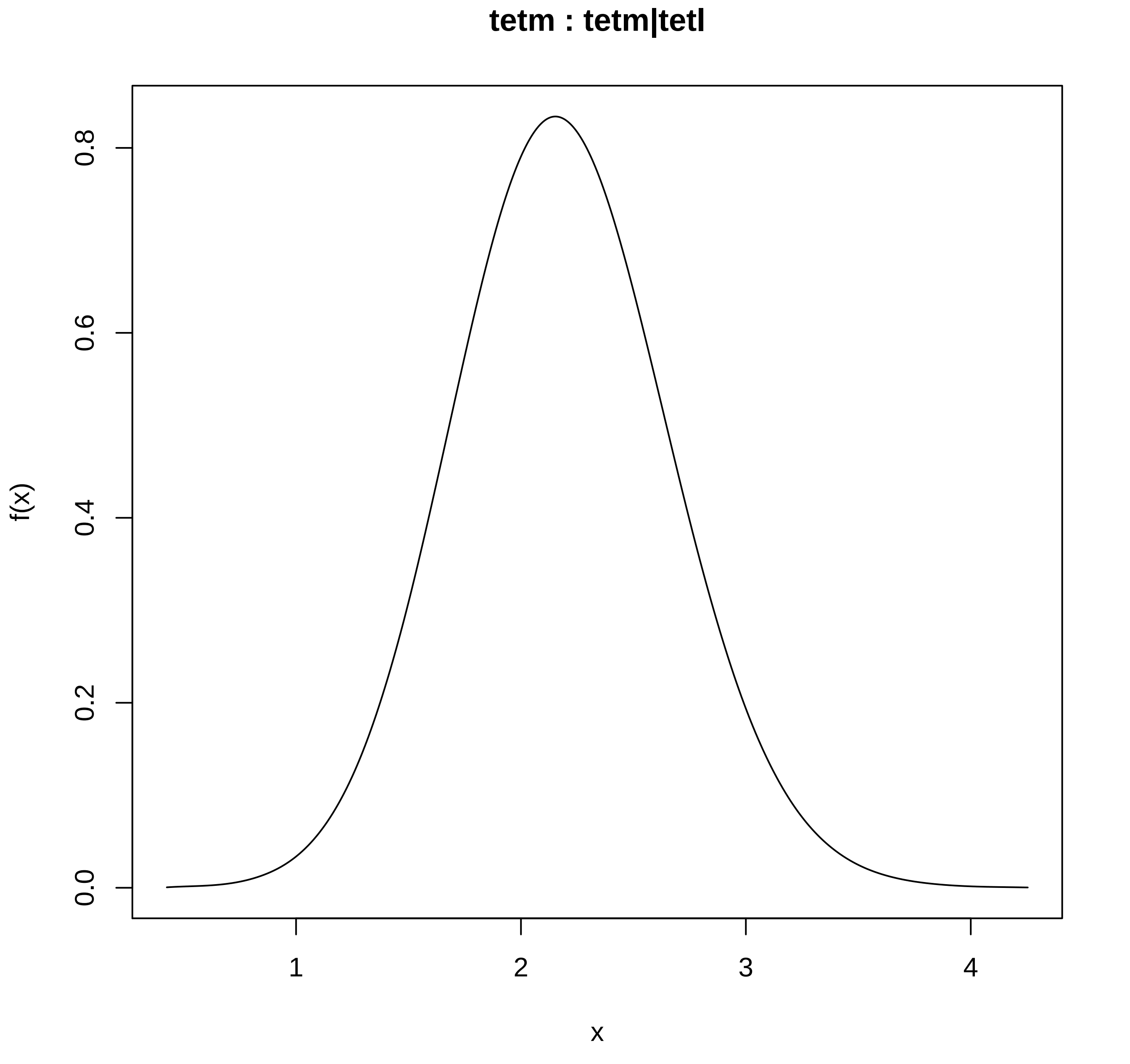

tetm : tetm|tetl
f(x)
0.0
0.2
0.4
0.6
0.8
1
2
3
4
x

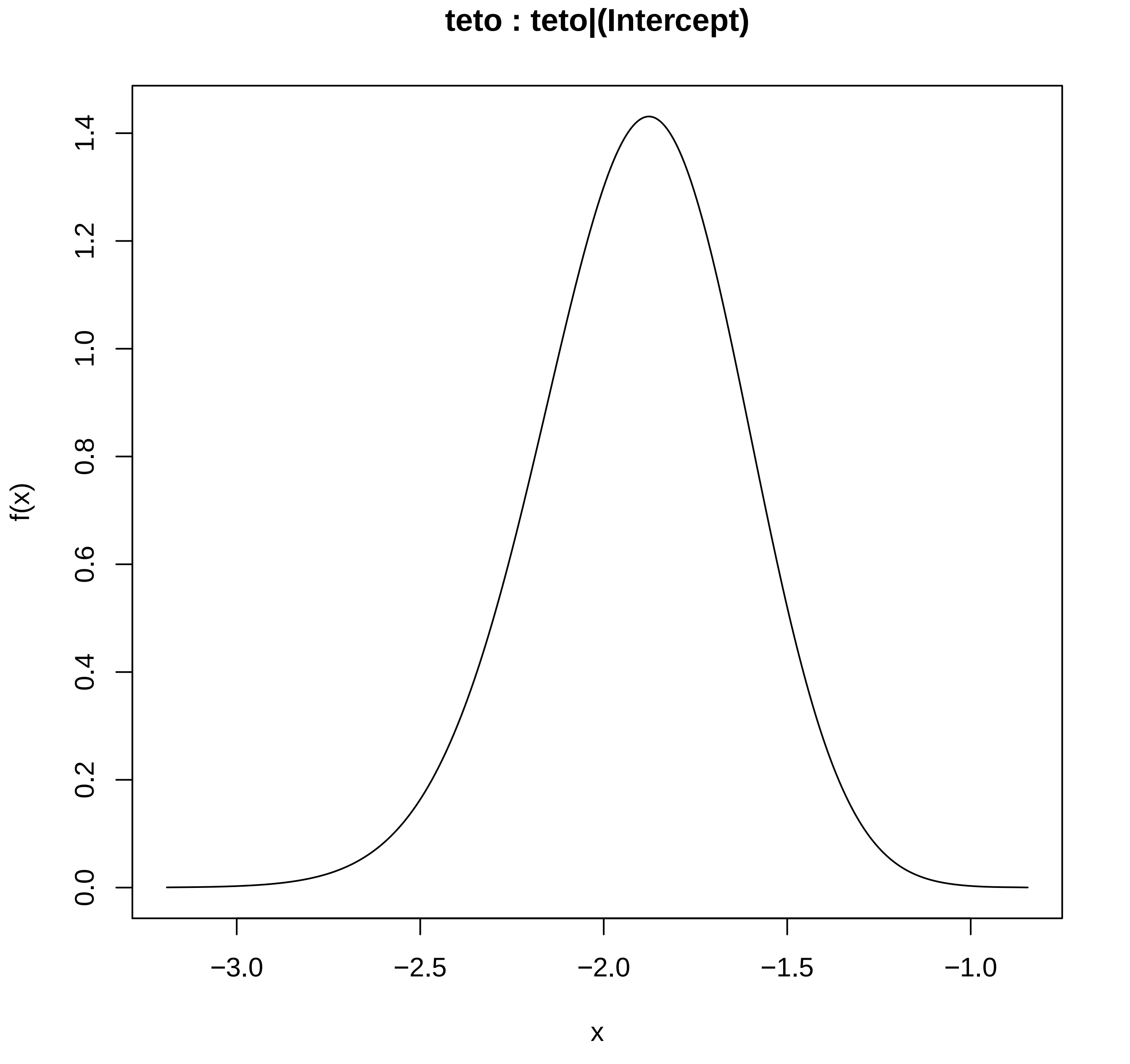

teto : teto|(Intercept)
f(x)
0.0
0.2
0.4
0.6
0.8
1.0
1.2
1.4
−3.0
−2.5
−2.0
−1.5
−1.0
x

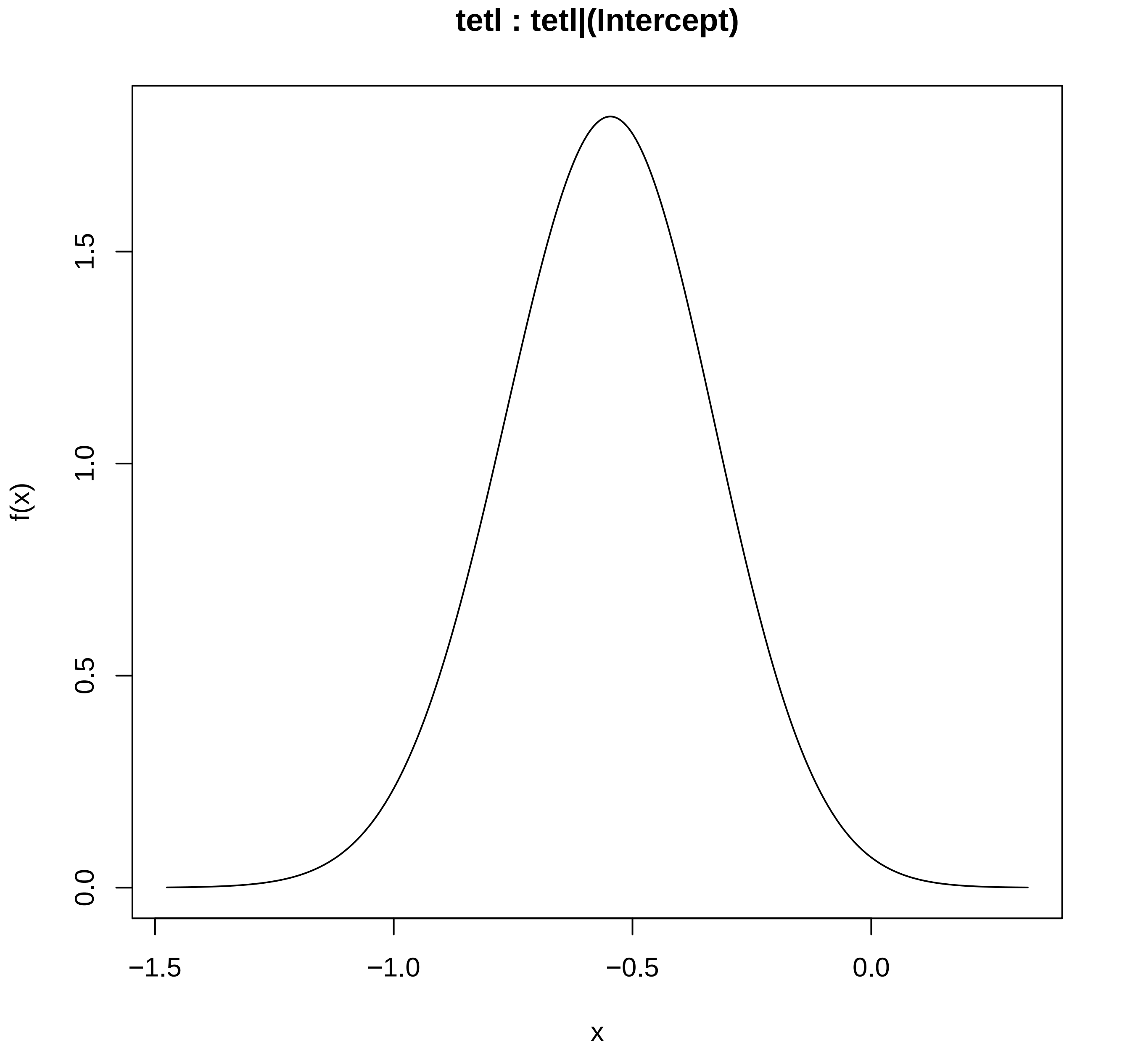
tetl : tetl|(Intercept)
f(x)
x
0.0
0.5
1.0
1.5
−1.5
−1.0
−0.5
0.0

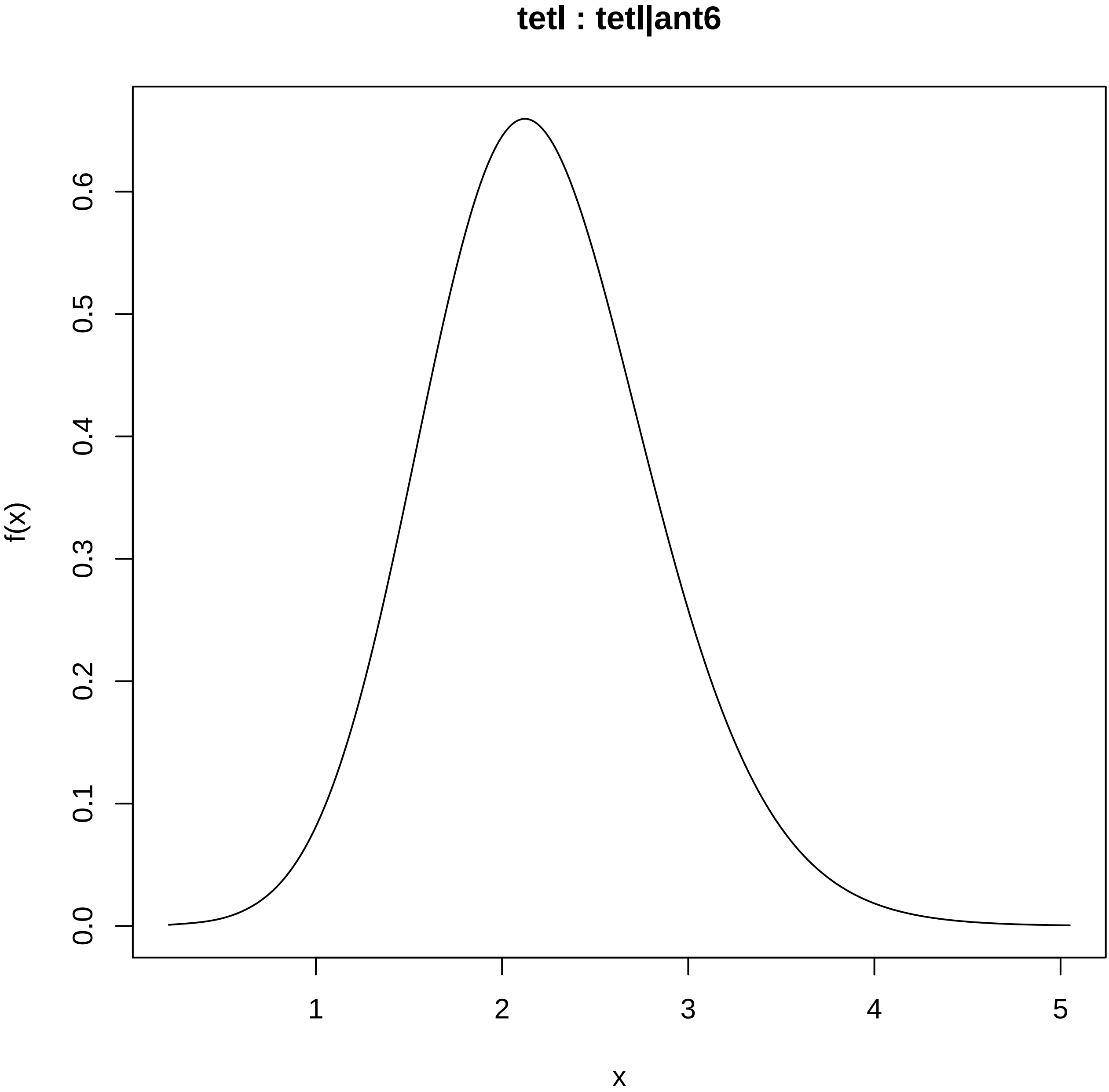

tetl : tetl|ant6
f(x)
0.0
0.1
0.2
0.3
0.4
0.5
0.6
1
2
3
4
5
x

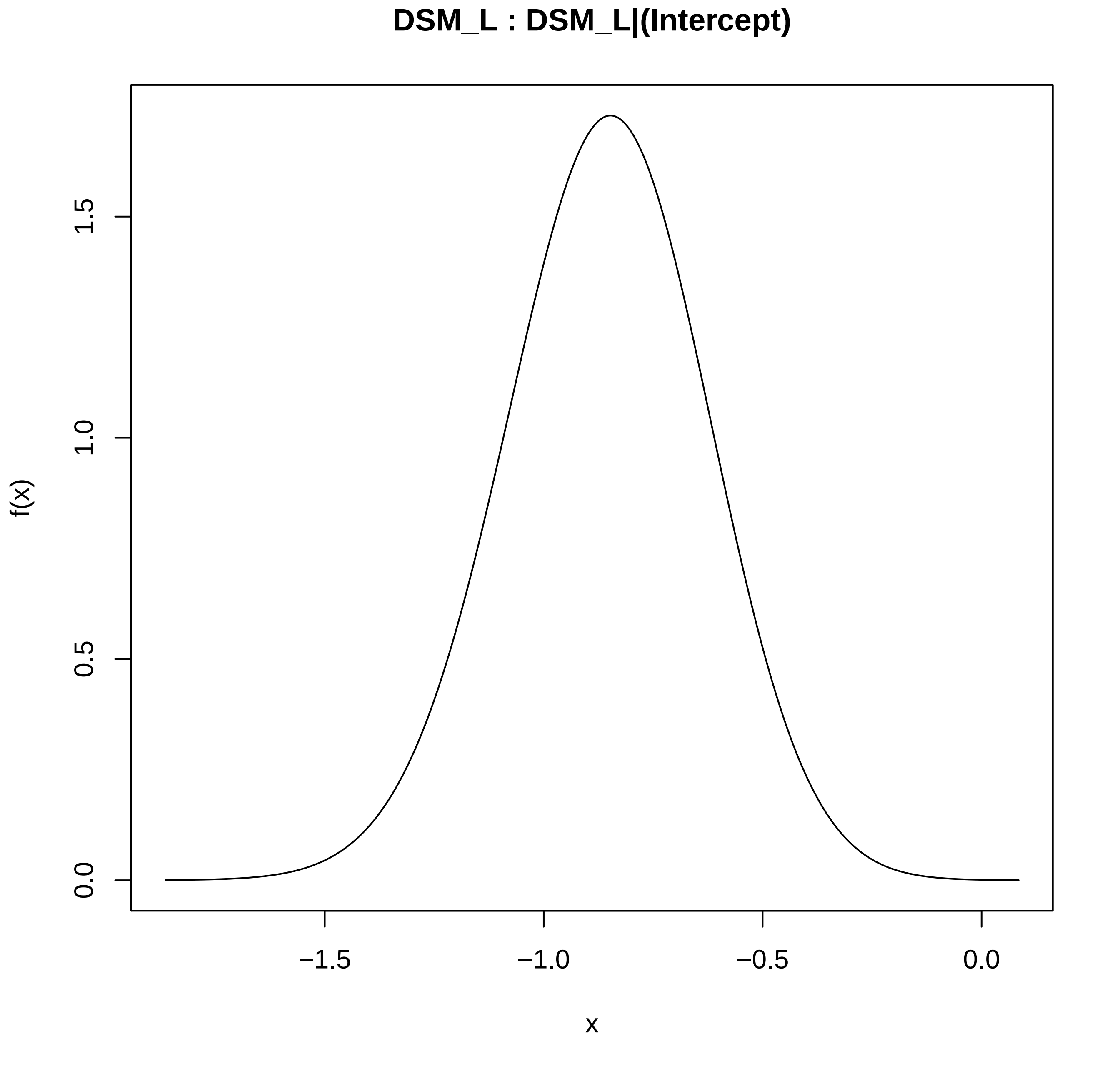
DSM_L : DSM_L|(Intercept)
f(x)
0.0
0.5
1.0
1.5
x
−1.5
−1.0
−0.5
0.0

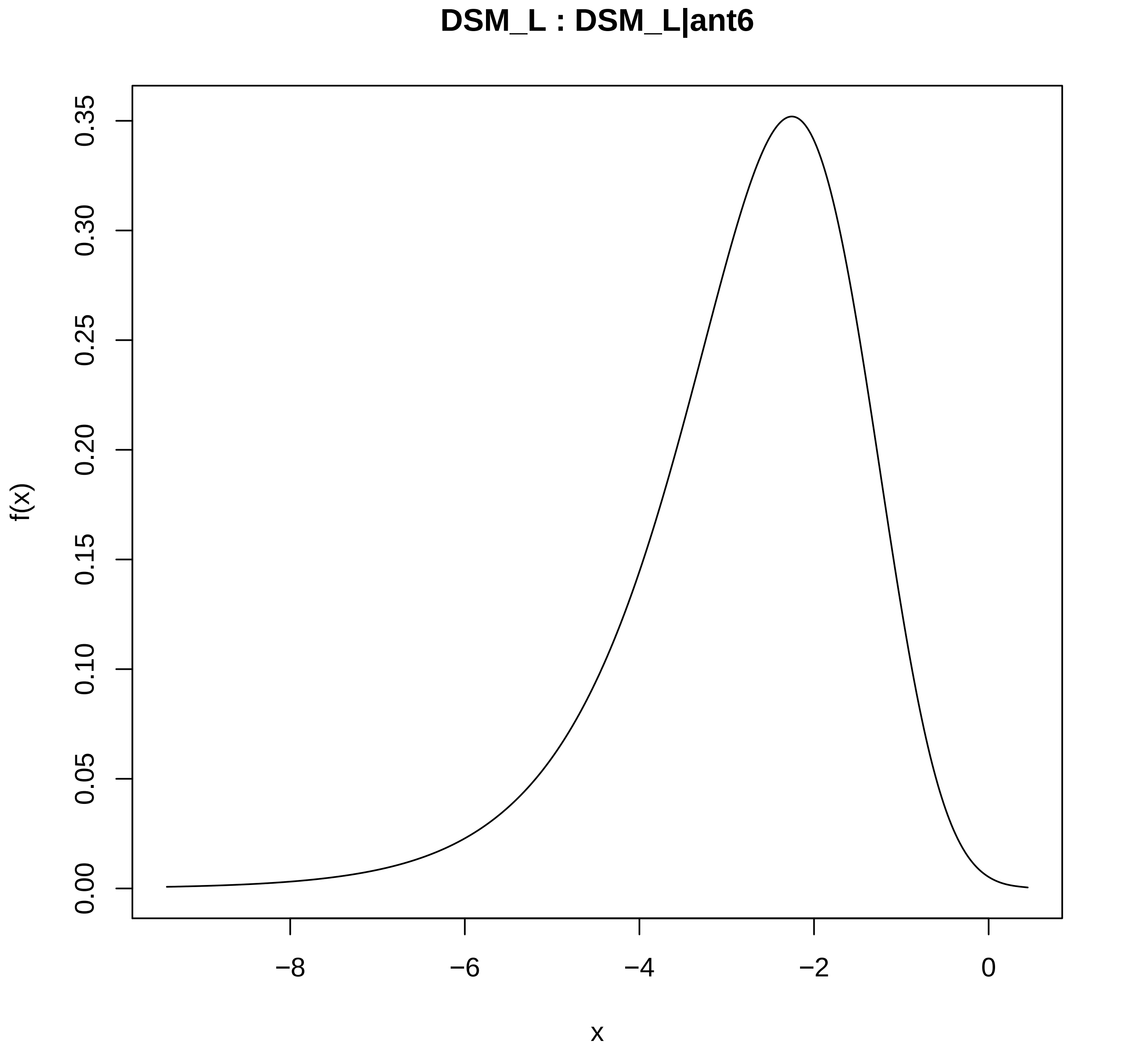
DSM_L : DSM_L|ant6
f(x)
0.00
0.05
0.10
0.15
0.20
0.25
0.30
0.35
−8
−6
−4
−2
0
x

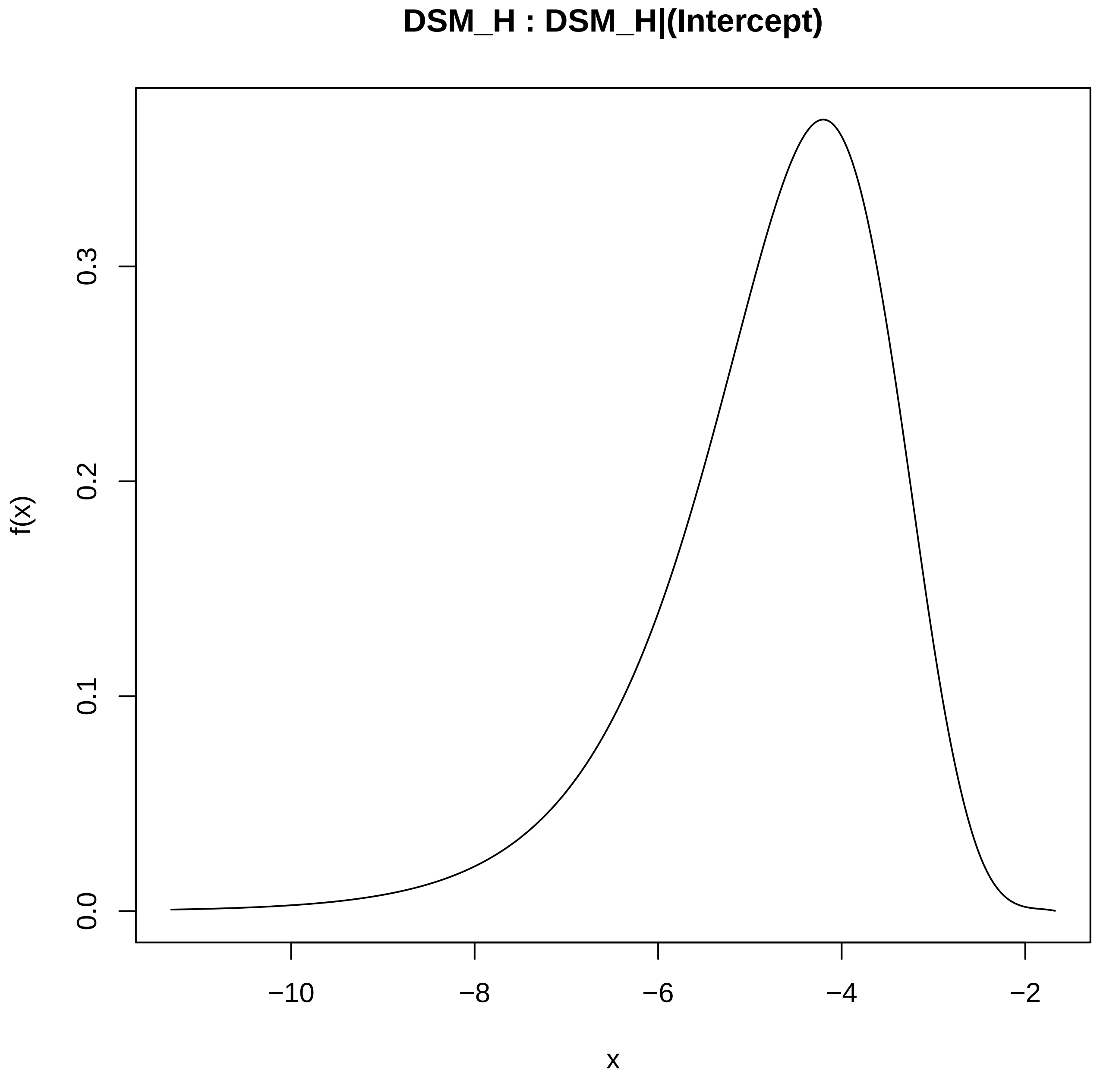
DSM_H : DSM_H|(Intercept)
f(x)
0.0
0.1
0.2
0.3
−10
−8
−6
−4
−2
x

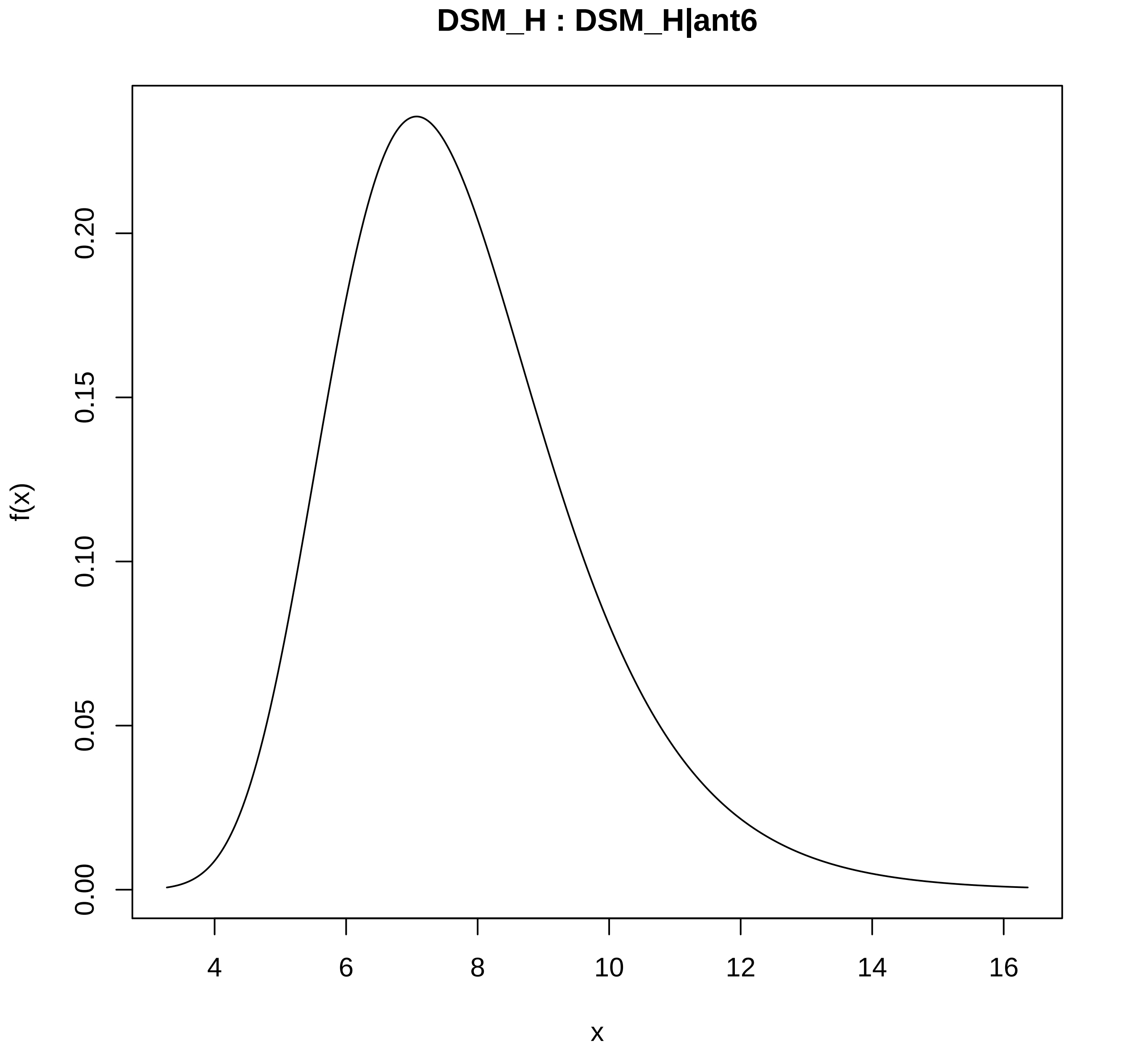
DSM_H : DSM_H|ant6
f(x)
0.00
0.05
0.10
0.15
0.20
4
6
8
10
12
14
16
x

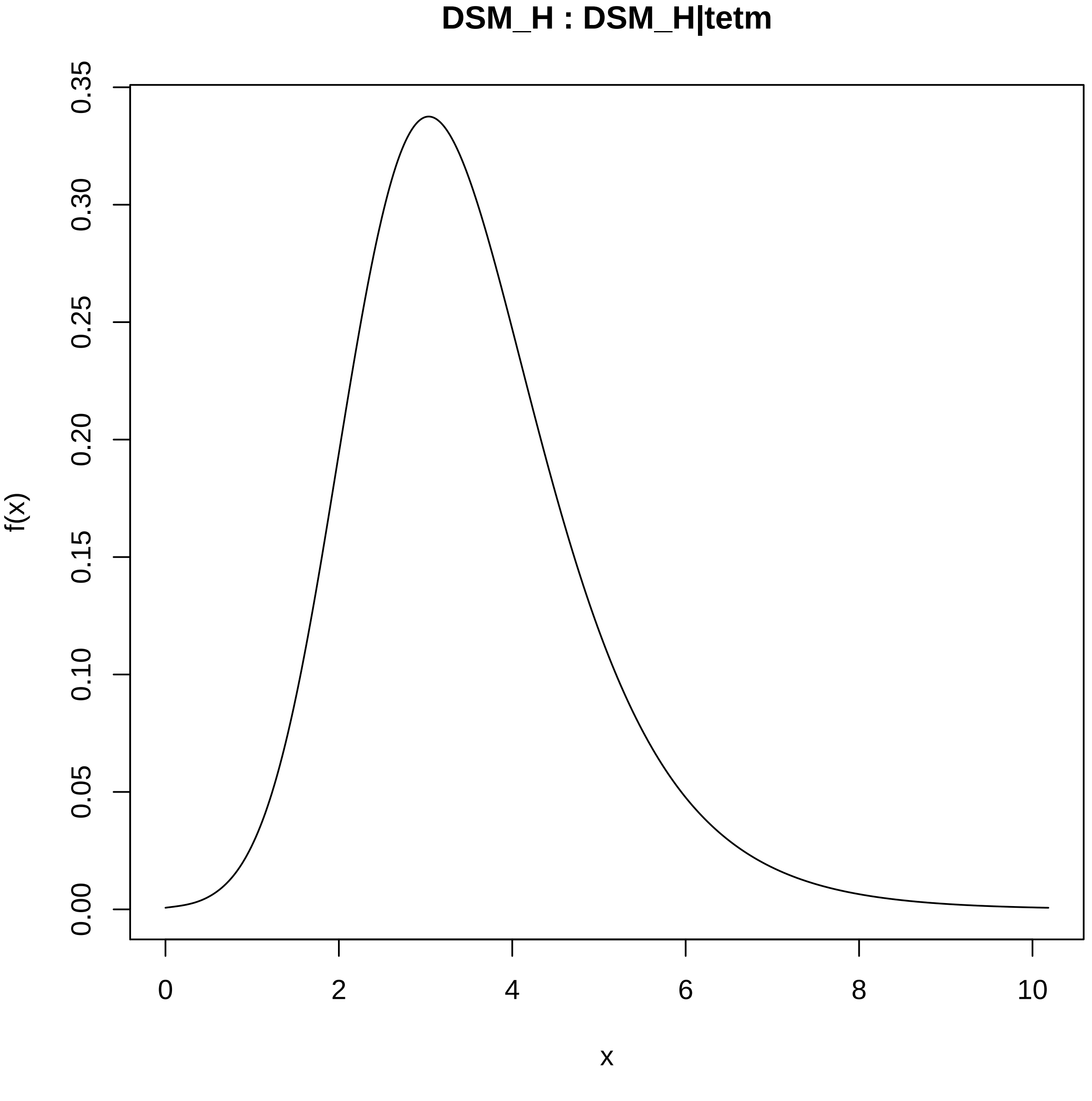
DSM_H : DSM_H|tetm
0.00
0.05
0.10
0.15
0.20
0.25
0.30
0.35
f(x)
0
2
4
6
8
10
x

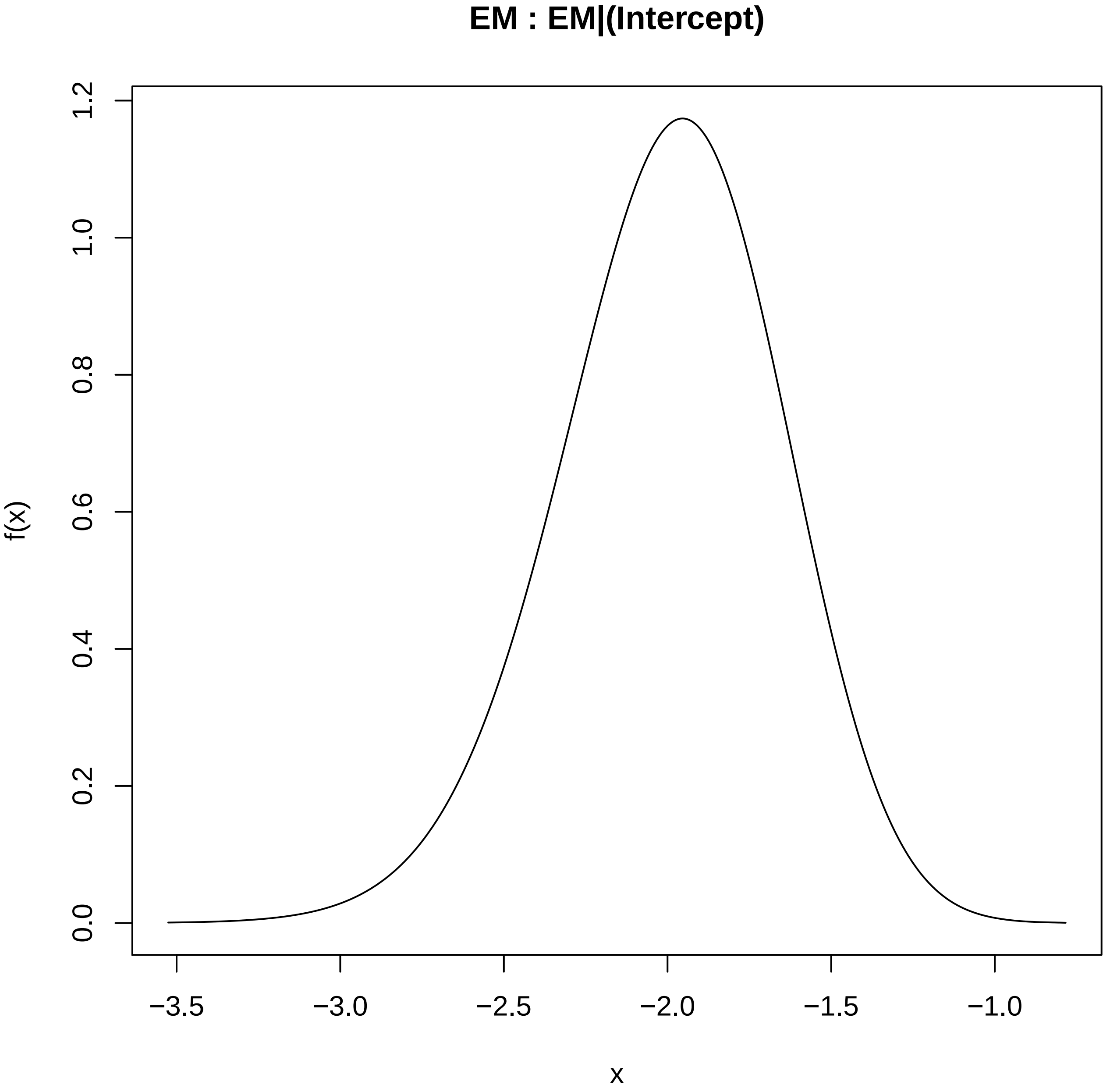

EM : EM|(Intercept)
f(x)
x
0.0
0.2
0.4
0.6
0.8
1.0
1.2
−3.5
−3.0
−2.5
−2.0
−1.5
−1.0

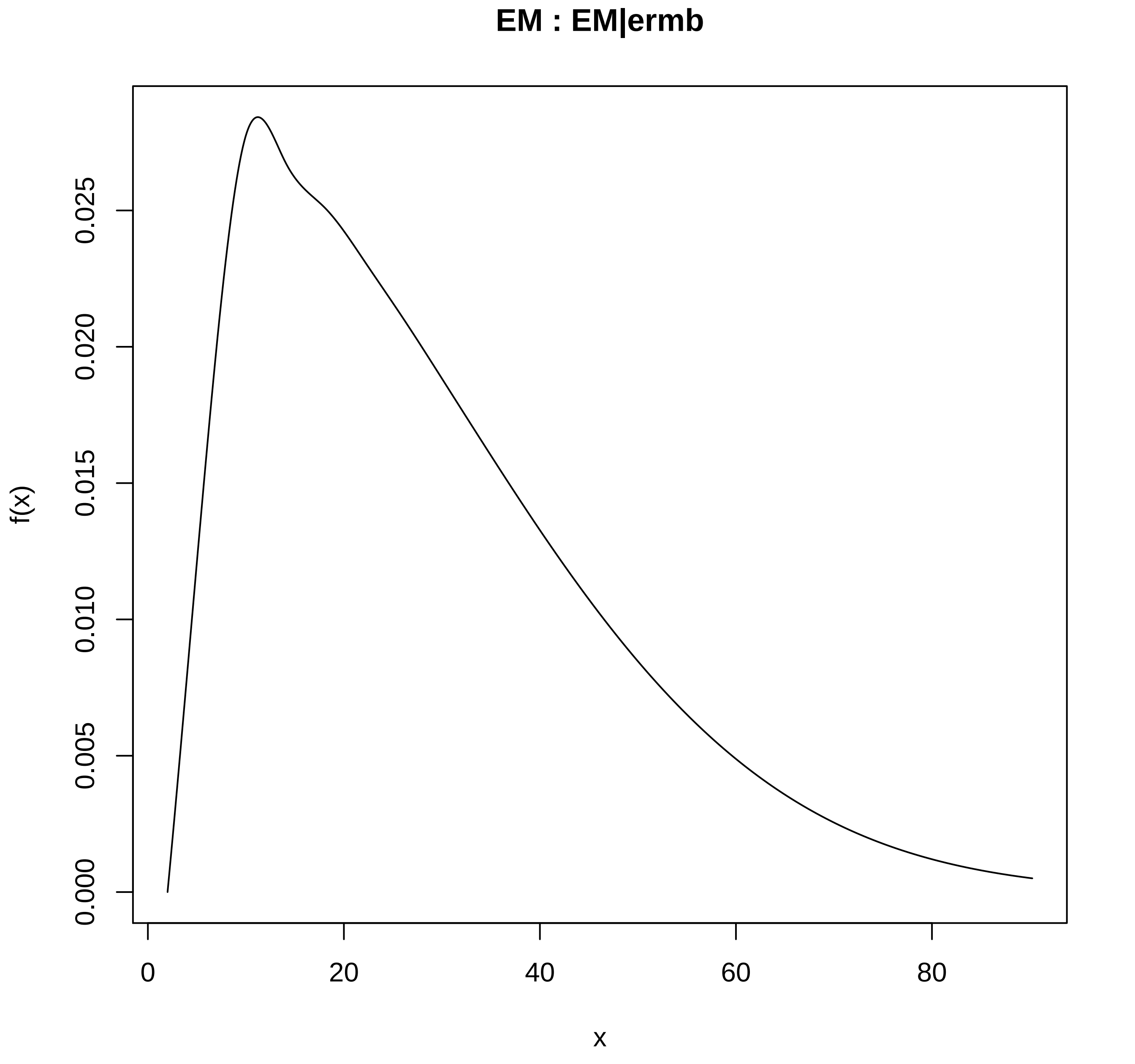
EM : EM|ermb
f(x)
0.000
0.005
0.010
0.015
0.020
0.025
0
20
40
60
80
x

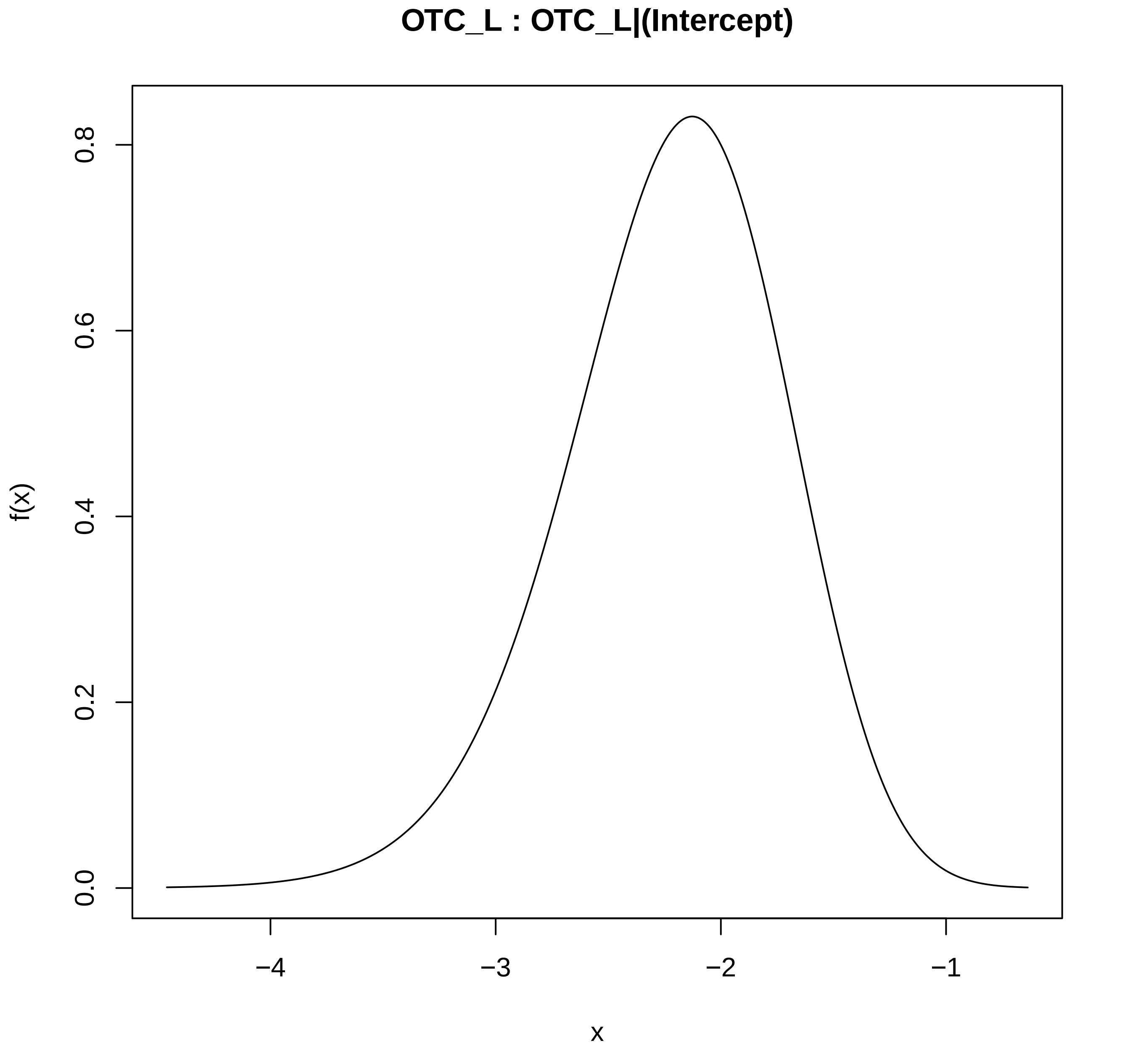
OTC_L : OTC_L|(Intercept)
f(x)
0.0
0.2
0.4
0.6
0.8
−4
−3
−2
−1
x

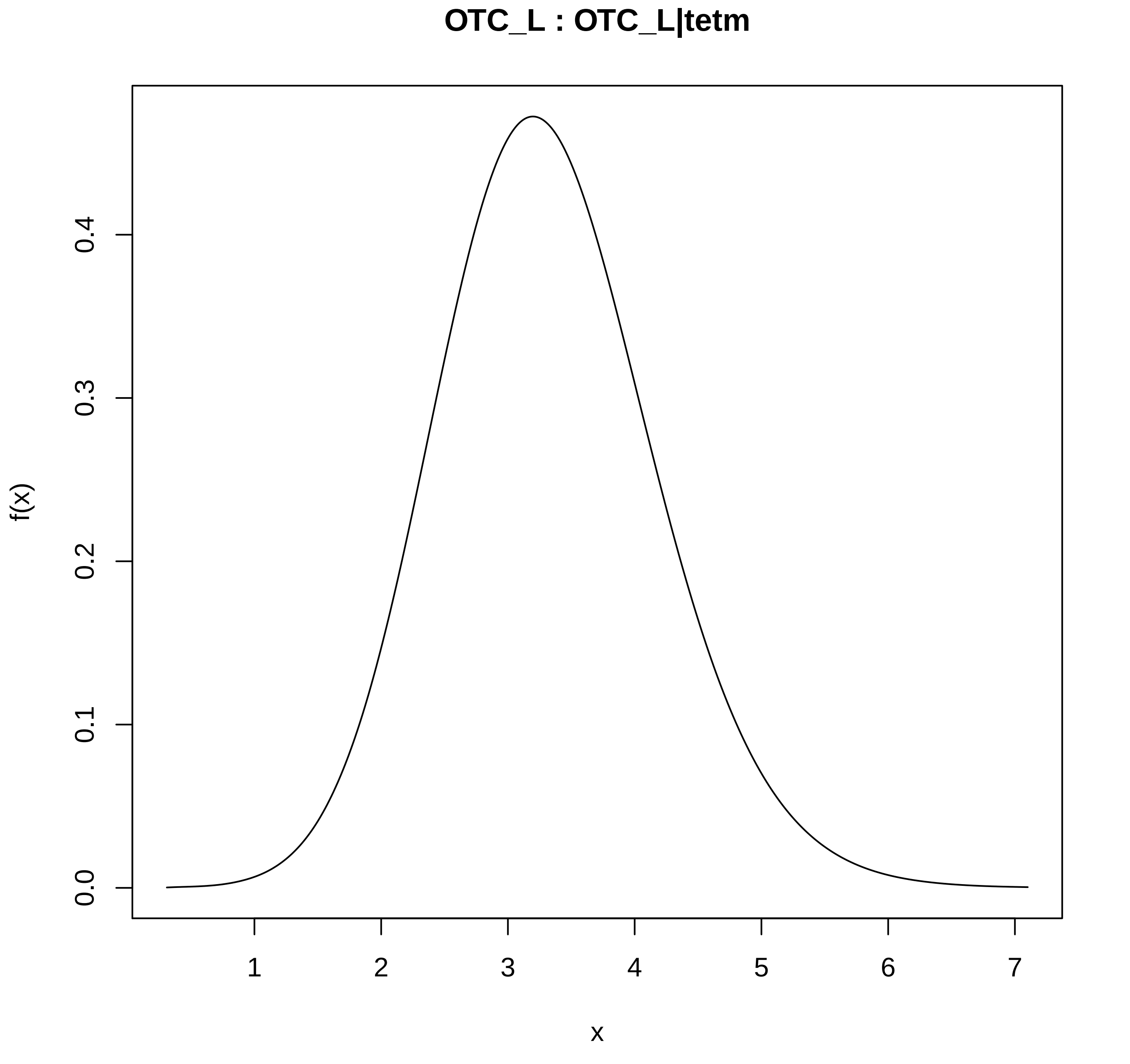
OTC_L : OTC_L|tetm
f(x)
0.0
0.1
0.2
0.3
0.4
1
2
3
4
5
6
7
x

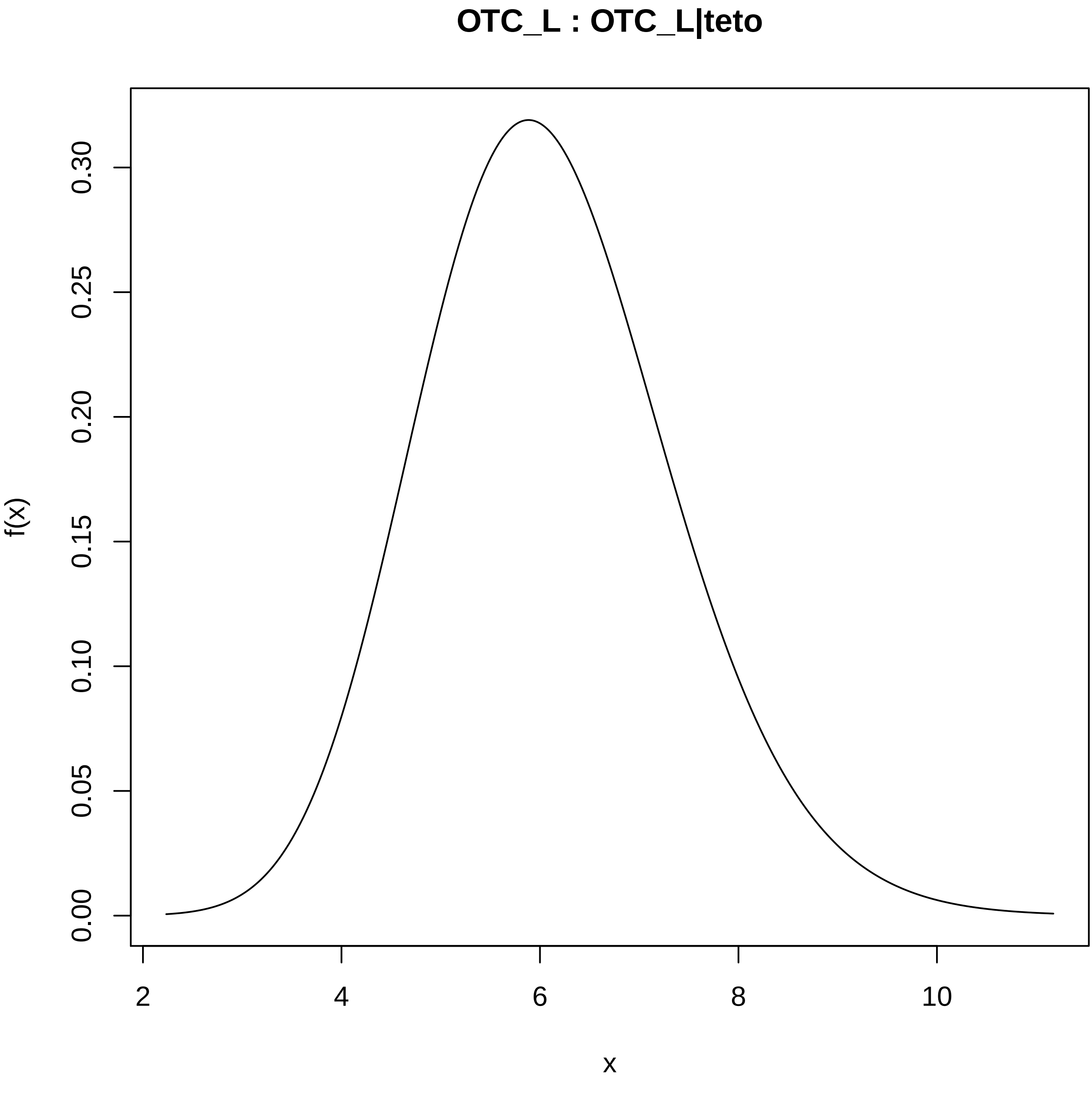
OTC_L : OTC_L|teto
f(x)
0.00
0.05
0.10
0.15
0.20
0.25
0.30
2
4
6
8
10
x

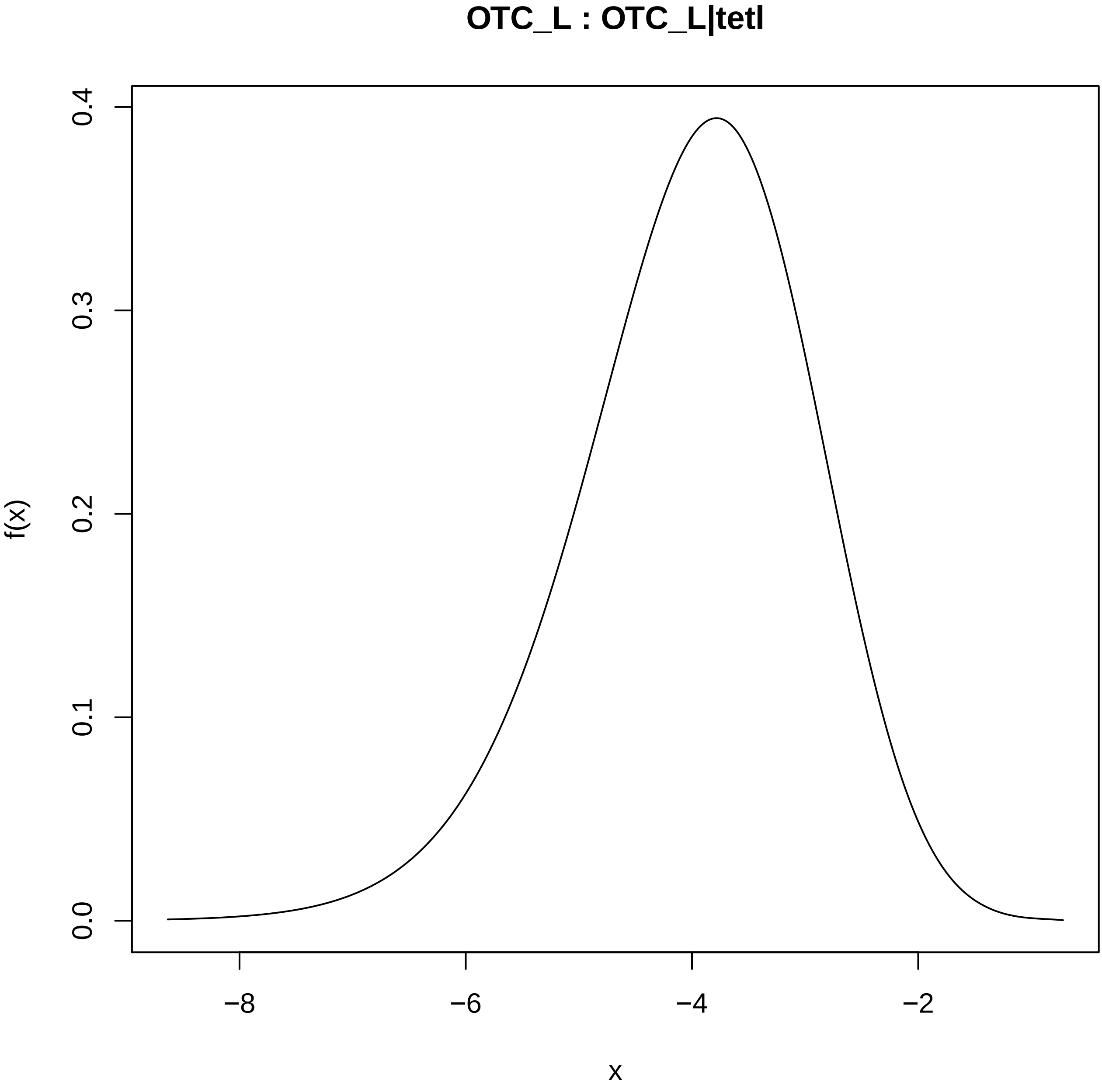
OTC_L : OTC_L|tetl
f(x)
0.0
0.1
0.2
0.3
0.4
−8
−6
−4
−2
x

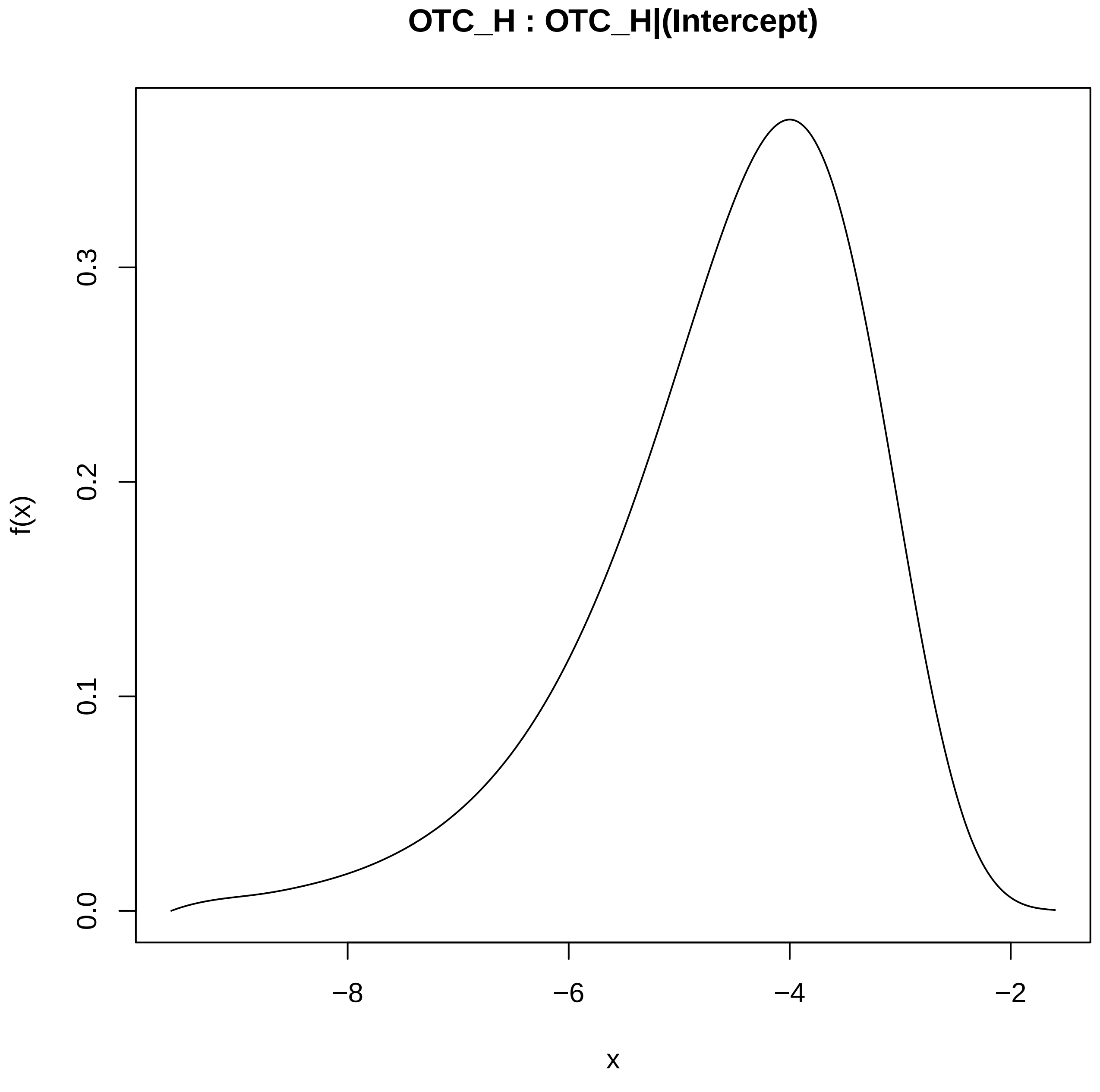
OTC_H : OTC_H|(Intercept)
f(x)
0.0
0.1
0.2
0.3
x
−8
−6
−4
−2

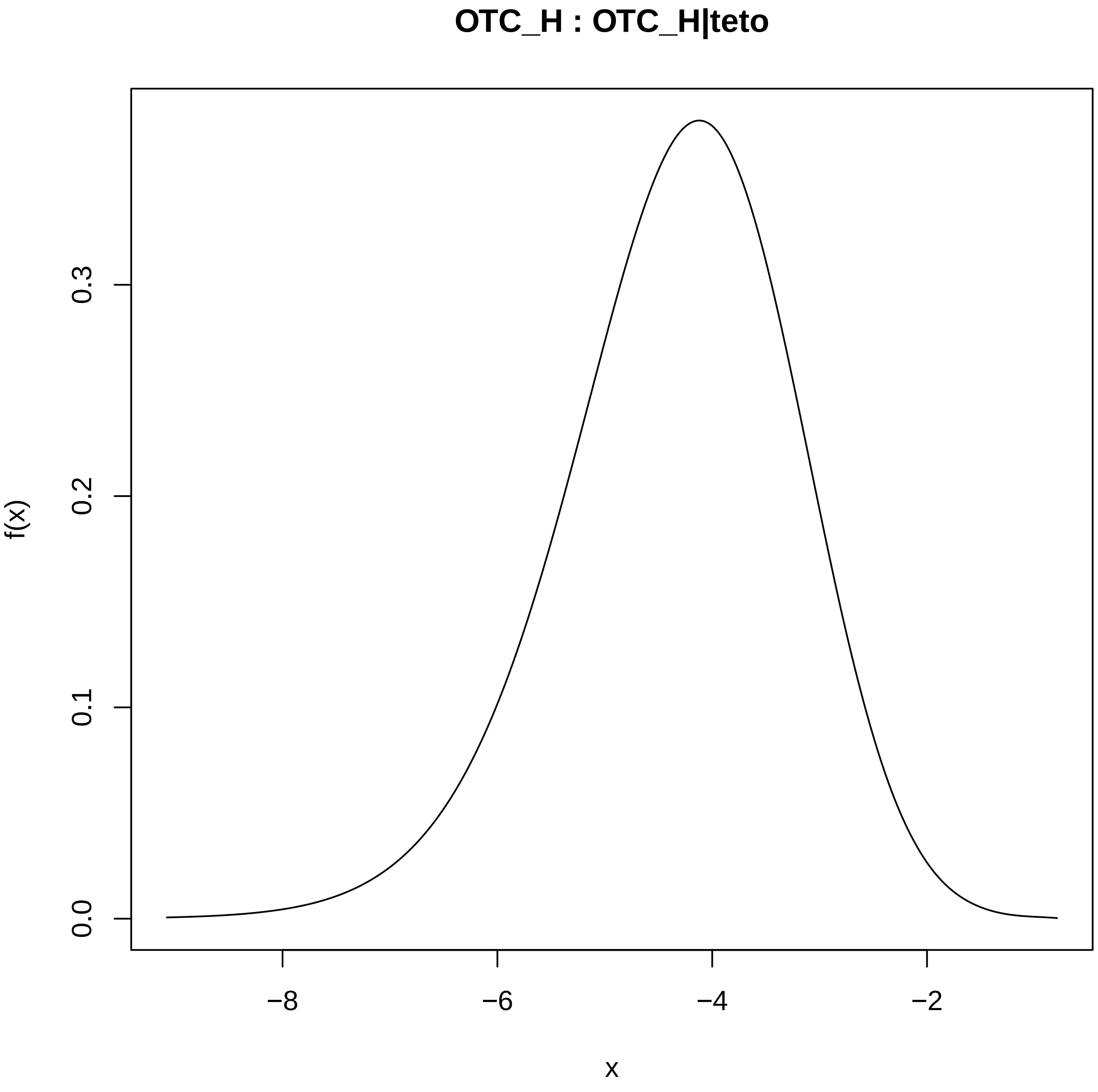
OTC_H : OTC_H|teto
0.0
0.1
0.2
0.3
f(x)
−8
−6
−4
−2
x

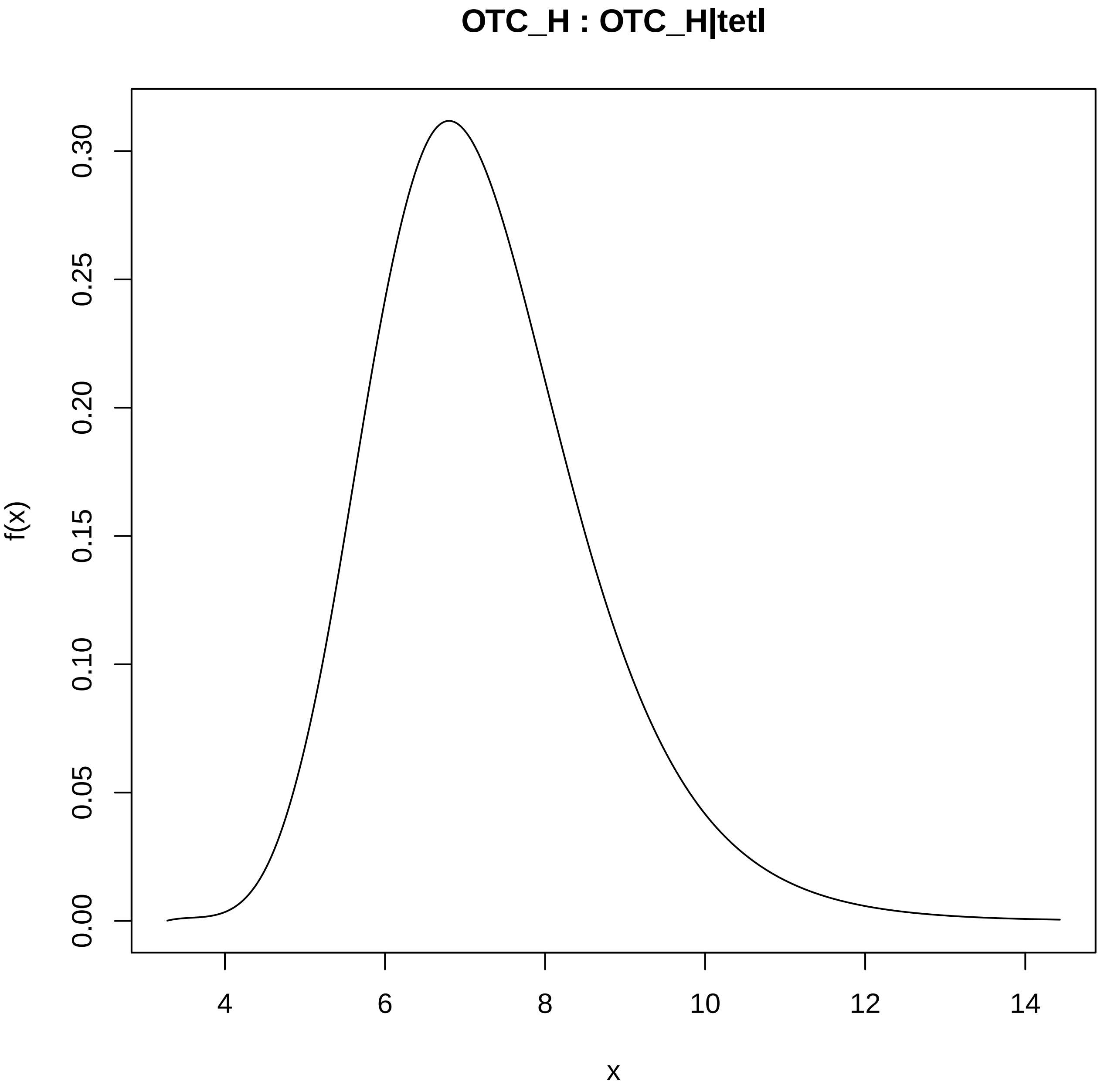
OTC_H : OTC_H|tetl
f(x)
0.00
0.05
0.10
0.15
0.20
0.25
0.30
4
6
8
10
12
14
x
