# Supplementary material for: Unraveling Antimicrobial Resistance Genes and Phenotype Patterns among Enterococcus faecalis Isolated from Retail Chicken Products in Japan
Source: PLoS One. 2015 Mar 17;10(3):e0121189. doi: 10.1371/journal.pone.0121189 (PMC4363150; doi:10.1371/journal.pone.0121189)
Supplement: S1 Table — (DOCX) [file pone.0121189.s005.docx]

**S1 Table. Frequency of arc appearances in 10,000 bootstrappings.**

| **row.names** | **ant6** | **aph3** | **ermb** | **tetm** | **teto** | **tetl** | **DSM_L** | **DSM_H** | **EM** | **OTC_L** | **OTC_H** |
| --- | --- | --- | --- | --- | --- | --- | --- | --- | --- | --- | --- |
| ant6 | 0 | 2884 | 2136^a^ | 68 | 281 | 2953 | 0 | 0 | 0 | 0 | 0 |
| aph3 | **6965** | 0 | **7571** | 124 | 884 | 1444 | 0 | 0 | 0 | 0 | 0 |
| ermb | 187^a^ | 2429 | 0 | 97 | **5756** | **6996** | 0 | 0 | 0 | 0 | 0 |
| tetm | 61 | 72 | 187 | 0 | **5760** | **7802** | 0 | 0 | 0 | 0 | 0 |
| teto | 308 | 566 | 1120 | 1640 | 0 | 74 | 0 | 0 | 0 | 0 | 0 |
| tetl | **5410** | 202 | 2876 | 1789 | 290 | 0 | 0 | 0 | 0 | 0 | 0 |
| DSM_L | **6520** | 286 | 78 | 55 | 258 | 99 | 0 | 0 | 0 | 0 | 0 |
| DSM_H | **10000** | 468 | 162 | **9315** | 829 | 239 | 0 | 0 | 0 | 0 | 0 |
| EM | 522 | 245 | **10000** | 525 | 1040 | 415 | 0 | 0 | 0 | 0 | 0 |
| OTC_L | 155 | 112 | 139 | **8735** | **9965** | **8905** | 0 | 0 | 0 | 0 | 0 |
| OTC_H | 740 | 595 | 681 | 838 | **5935** | **10000** | 0 | 0 | 0 | 0 | 0 |

Numbers in bold indicate that the arc appeared in more than 50% of bootstrappings. Frequency of arcs recovered was counted by collapsing the direction of arcs. The most supported arc direction was, however, used when marginal log odds were calculated.

^a^ Arc between *tet*(O) and *tet*(L) appeared more than 50%, however, this arc did not exist in the initial model and hence excluded.
