# Supplementary material for: Unraveling Antimicrobial Resistance Genes and Phenotype Patterns among Enterococcus faecalis Isolated from Retail Chicken Products in Japan
Source: PLoS One. 2015 Mar 17;10(3):e0121189. doi: 10.1371/journal.pone.0121189 (PMC4363150; doi:10.1371/journal.pone.0121189)
Supplement: S1 Text — (DOCX) [file pone.0121189.s006.docx]

#R code to identify the initial DAG using exact method #data can be found in FileS1.

#Comprehensive R code can be also found at http://www.r-bayesian-networks.org/case-study-one

library(abn)

#Specify prohibited linkage (i.e. between resistance phenotype and arcs from phenotype to resistance #gene)

banned<-matrix(c(

0,0,0,0,0,0,1,1,1,1,1, # ant6

0,0,0,0,0,0,1,1,1,1,1, # aph3

0,0,0,0,0,0,1,1,1,1,1, # ermB

0,0,0,0,0,0,1,1,1,1,1, # tetM

0,0,0,0,0,0,1,1,1,1,1, # tetO

0,0,0,0,0,0,1,1,1,1,1, # tetL

0,0,0,0,0,0,1,1,1,1,1, # DSM_L

0,0,0,0,0,0,1,1,1,1,1, #DSM_H

0,0,0,0,0,0,1,1,1,1,1, #EM

0,0,0,0,0,0,1,1,1,1,1, #OTC_L

0,0,0,0,0,0,1,1,1,1,1) #OTC_H

, byrow=T, ncol=11)

colnames(banned)<-rownames(banned)<-names(data)

#Retain links (here no arcs are forced into the model)

retain<-matrix(c(

0,0,0,0,0,0,0,0,0,0,0, # ant6

0,0,0,0,0,0,0,0,0,0,0, # aph3

0,0,0,0,0,0,0,0,0,0,0, # ermB

0,0,0,0,0,0,0,0,0,0,0,# tetM

0,0,0,0,0,0,0,0,0,0,0, #tetO

0,0,0,0,0,0,0,0,0,0,0, #tetL

0,0,0,0,0,0,0,0,0,0,0, #DSM_L

0,0,0,0,0,0,0,0,0,0,0, #DSM_H

0,0,0,0,0,0,0,0,0,0,0,# EM

0,0,0,0,0,0,0,0,0,0,0, #OTC_L

0,0,0,0,0,0,0,0,0,0,0 #OTC_H

), byrow=T,ncol=11)

colnames(retain)<-rownames(retain)<-names(data)

#Specify distributions for 11 parameters – all binary variables and hence follow binomial distribution

mydist<-list(ant6="binomial",aph3="binomial",ermb="binomial",tetm="binomial",teto="binomial",tetl="binomial", DSM_L="binomial", DSM_H="binomial",EM="binomial", OTC_L="binomial", OTC_H="binomial")

#Calculate marginal log-likelihood and compare them when maximum number of edges are increased

mycache1<-buildscorecache(data.df=data, data.dists=mydist,

dag.banned=banned, dag.retained=retain, max.parents=1);

mp.dag<-mostprobable(score.cache=mycache1);

mlik1<-fitabn(dag.m=mp.dag, data.df=data, data.dists=mydist)$mlik; #give marginal log-likelihood

#/*repeat this increasing the number of max.parents 1 by 1 until marginal log-likelihood does not #increase. Here we found max.par = 3 was sufficient.*/

#Specify maximum number of edges per parent node

max.par<-3

mycache<-buildscorecache(data.df=data, data.dists=mydist,

dag.banned=banned, dag.retained=retain, max.parents=3);

#Exact search method to identify most probable DAG

mp.dag<-mostprobable(score.cache=mycache);

initialDAG<-fitabn(dag.m=mp.dag,data.df=data,data.dists=mydist,create.graph=TRUE);

#Draw initial DAG

plot(initialDAG$graph);
